# Neuromodulation Improves Stress Urinary Incontinence-Like Deficits in Female Rabbits

**DOI:** 10.1109/OJEMB.2024.3408454

**Published:** 2024-06-03

**Authors:** F. S. Rahman, Z. Yousuf, F. Castelán, M. Martínez-Gómez, Y. M. Akay, P. Zimmern, M. Akay, M. I. Romero-Ortega

**Affiliations:** ^1^ University of Houston 14743 Houston TX 77004 USA; ^2^ University of Arizona 8041 Tucson AZ 85721 USA; ^3^ Centro Tlaxcala de Biología de la ConductaUniversidad Autónoma de Tlaxcala 27776 Tlaxcala 19 México; ^4^ Universidad Nacional Autonoma de México, Unidad Foránea TlaxcalaInstituto de Investigaciones Biomédicas 54448 Tlaxcala 19 México; ^5^ University of Texas Southwestern Medical Center 12334 Dallas TX 75390 USA

**Keywords:** Bioelectronics, electrical stimulation, pelvic floor disorders, pelvic innervation, neural interfaces

## Abstract

*Objective:* Stress urinary incontinence (SUI) affects a third of the female population and is characterized by involuntary urine leakage during abdominal efforts such as sneezing, laughing, or coughing. Acute neuromodulation of the bulbospongiosus nerve (BsN) was shown to increase bladder efficiency in aged and multiparous rabbits. This study investigates the efficacy of sub-chronic BsN neuromodulation in alleviating SUI-like deficits in mature multiparous rabbits, characterized by increased urine leakage and reduced leak point pressure*. Results:* Using the voiding spot assay, we observed a 40% reduction in urine leakage events after 30 days of BsN stimulation, which correlated with a 60% increase in daily micturition volume, a 10-fold increase in voided volume, and improvements in voiding efficiency and leak point pressure compared to negative control animals. *Conclusion:* In multiparous rabbits, BsN neuromodulation improves important SUI-like metrics including bladder capacity and urethral closure, supporting the use of this bioelectronic modality as treatment for SUI.

## I. Introduction

Stress urinary incontinence (SUI) is a condition characterized by involuntary urine leakage during sudden increases in intra-abdominal pressure, such as during jumping, sneezing, laughing, or coughing [1]. Pelvic floor muscles (PFMs) provide mechanical support to pelvic organs and proximally wrap around the urethra as part of the perineal complex, assisting in urethral closure [2]. These muscles act as a secondary sphincter during sudden increases in abdominal pressure, assisting the external urinary sphincter (EUS) and contributing to the “guardian reflex” to prevent leakage [3]. Weakened PFMs contribute to SUI, which affect approximately 20–35% of adult women, and result from several factors, including pregnancy, parity, and aging [4], [5], [6], [7], [8], [9], [10].

Animals models of SUI include aging and/or multiparous female rabbits, which show anatomical and physiological signs of weakened PFM, deficient urethral closure, and reduced bladder capacity. [11], [12], [13], [14], [15], [16], [17]. This animal model also shows partial nerve damage of the pelvic and perineal nerves including the pubococcygeus nerve (PcN) and bulbospongiosus nerve (BsN) [18], [19]. We previously reported that acute electrical stimulation (ES) of the BsN in multiparous rabbits effectively recruits the bulbospongiosus muscle (BsM), increasing the maximum urethral closure pressure and partially reversing the deficits on bladder efficiency and urethral closure [18], [19]. However, the long-term efficacy of this therapy had not been previously investigated.

In this study we developed a miniaturized implantable neural stimulator and evaluated the effect of a 30-day wireless neuromodulation of the BsN in mature multiparous (MM) female rabbits. SUI-like deficits such as spontaneous urine leakage, daily voided volume, and leak point pressure were investigated. We observed a decrease in SUI-like deficits, supporting the use of this bioelectronic approach as a potential treatment for SUI.

## II. Results

### A. Wireless Neural Stimulator Evokes BsM Contraction

**Figure 1.**
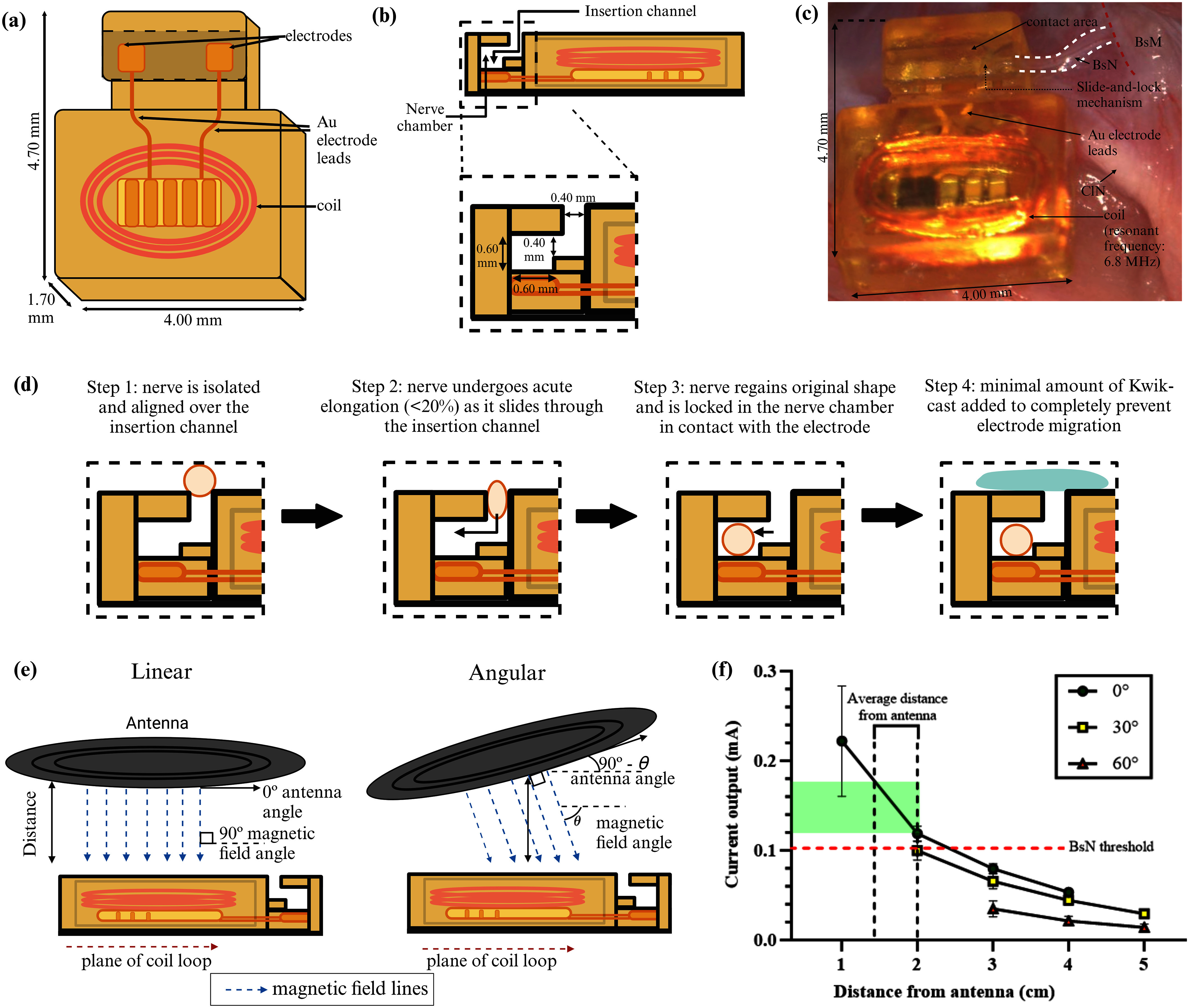
wNClip implantation and function. (a), (b) Device schematic, and (c) picture of device on the BsN. (d) Schematic of the “slide-and-lock” mechanism of wNClip implantation. (e) Angular test for powering the wNClip using the external antenna. (f) Current output as a function of antenna distance and angle of 29 wNClip devices. Shaded green area shows targeted current output.

Twenty-nine miniature wireless NeuroClip (wNClip) stimulator devices (4.7 × 4.0 × 1.7 mm^3^; Fig. 1(a) and (b)) were fabricated by Juniper Biomedical (see supplemental materials 1.1). The device uses a slide-and-lock mechanism to clip onto the BsN. The wNClip is implanted by placing it underneath the target nerve, aligned over the insertion channel, and gently and briefly (i.e., for 15–30 s) lifting the device such that the nerve elongates, reducing its diameter (≤20%), and allowing it to pass through the narrow Z-shaped channel that is designed to prevent easy dislodgment. Once in the electrode chamber, releasing the pull on the nerve allows it to regain its original shape, thereby locking the nerve in place (Fig. 1(d)).

For stimulation, we use cathodic first bi-phasic electrical pulses powered by electromagnetic induction with an external antenna. A threshold current of 0.1 mA efficiently evoked the contraction of the BsM (see supplementary materials 1.2). The angular distance from the antenna sufficient to power the device and depolarize the BsN was evaluated. Current output was measured at 1 to 5 cm and angles of 0°, 30° and 60° (Fig. 1(e)). A maximum of 0.23 mA was measured at 1 cm from the antenna. Effective BsM contraction was obtained at up to 2 cm and 30° angle form the antenna (Fig. 1(f)).

### B. BsN Stimulation Reduces Leak Events

**Figure 2.**
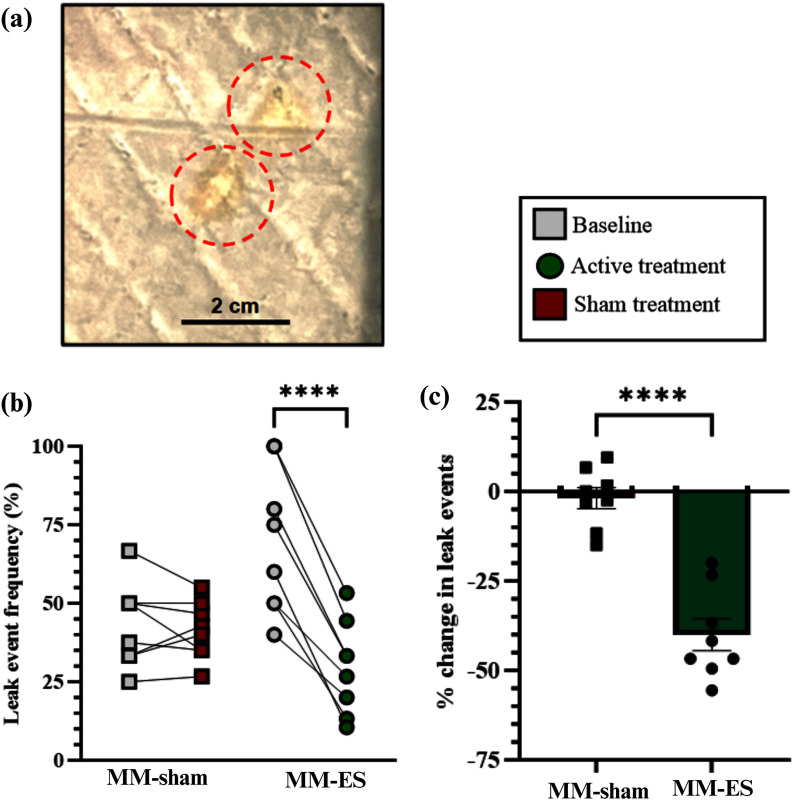
BsN ES reduces leak frequency. (a) Representative image of leak event identified on the absorbent pad. MM-ES group (n = 8) showed a significant decrease in leak event frequency, (b) between baseline and treatment periods, and (c) from the negative control MM-sham group (n = 8).

Leak events were determined as urine spots smaller than 3 mm on absorbent pads placed under the animal cage floor, monitored for 24-hours, three times per week [Fig. 2(a)]. A 2-week baseline period was established, followed by a 4-week BsN ES treatment period. This voiding spot assay (VSA) showed that young nulliparous (YN) rabbits (n=5) did not leak during the study, whereas mature multiparous sham rabbits (MM-sham, n=8) leaked 25–75% of the time. Sham ES (i.e., inactive electrodes or animals with no implant but exposed to the antenna pulsed electrical field) showed no change in the percentage of daily leak events. In contrast, mature multiparous rabbits that received neuromodulation treatment (MM-ES, n=8) showed a significant (p<0.0001) decrease in leak frequency, from 69.4 ± 8.2% at baseline, to 29.4 ± 5.2% during the treatment. This represented a 40.0 ± 4.5% reduction in leak frequency (Fig. 2(b) and (c)). The baseline values of both MM-ES and MM-sham group were comparable (p=0.09). These results demonstrated that BsM neuromodulation significantly reduced leak events, a metric associated with SUI.

### C. BsN ES Increases Micturition Volume

**Figure 3.**
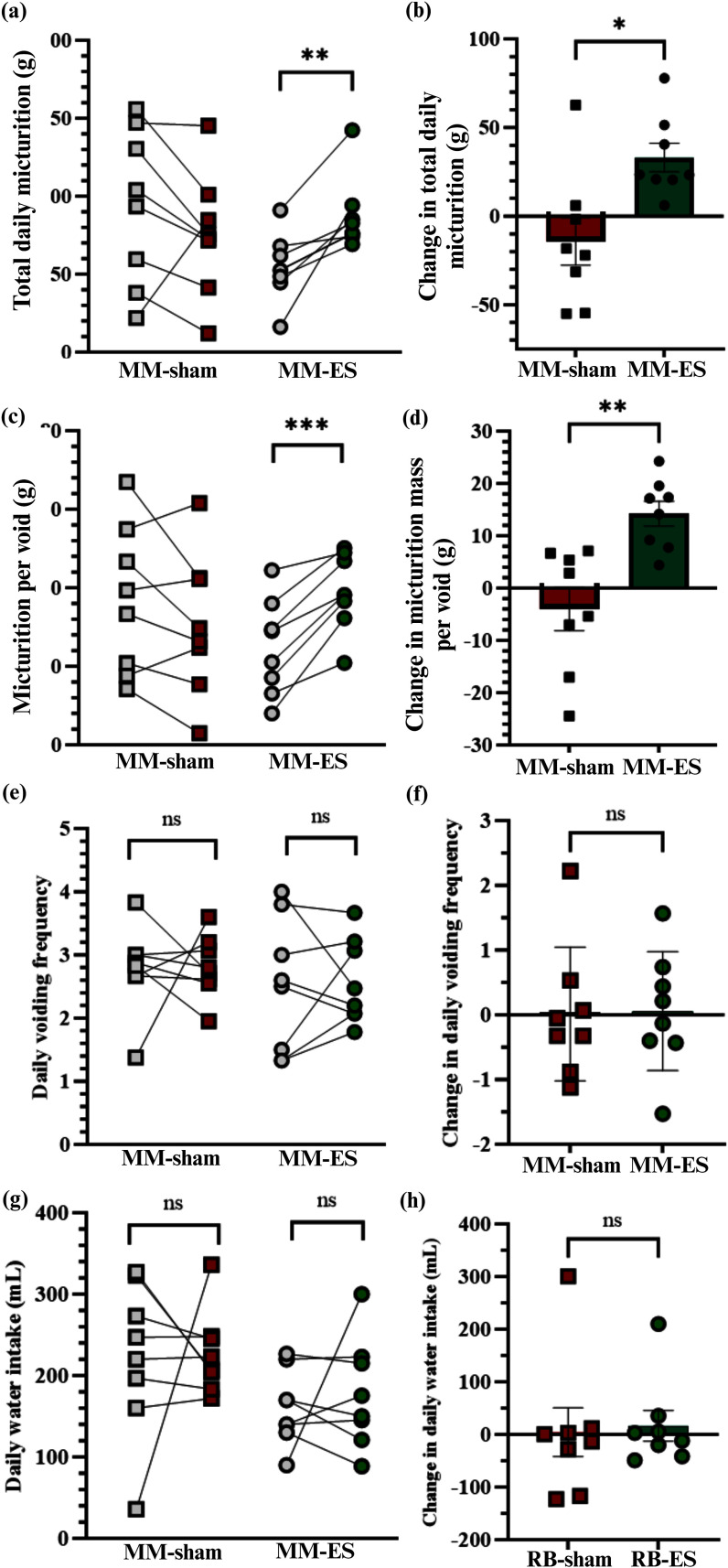
BsN ES achieved increased daily voided volume. MM-ES group showed increased (a and b) daily micturition; (c and d) increased average micturition per void. No significant differences were found between MM-ES and MM-sham groups for (e and f) voiding frequency; (g and h) daily water intake.

To evaluate if voided volume changed during micturition over time, we measured the pad weight 3 times a week and compared the values at baseline and during ES treatment. MM-ES animals showed a 33.1 ± 8.0 g (60.7%) increase in total daily voided volume compared to pre-treatment levels (54.5 ± 7.5g at baseline *vs.* 87.6 ± 8.3 g during treatment; p=0.01; n=8). Pad weight values in MM-sham animals did not change significantly (93.8 ± 17.7 g at baseline *vs.* 75.6 ± 13.9 g during treatment, p=0.2; Fig. 3(a) and (b)). The baseline values for the MM-ES and MM-sham groups were comparable (p= 0.2).

A similar trend was observed for the average micturition per void in MM-ES group, which showed a significant increase of 14.3 ± 2.4 g (p= 0.003) between baseline and stimulation periods (24.8 ± 4.3 g *vs*. 39.1 ± 3.5 g, p≤ 0.0005). Conversely, MM-sham rabbits showed similar values at baseline (36.8 ± 6.7 g) and treatment period (30.7 ± 6.4 g; p≤0.2) (Fig. 3(c) and (d)).

### D. BsN ES Does Not Affect Voiding Frequency or Daily Water Intake

We then evaluated if the increase in pad weight was due to an increase in the number of micturition events or an increased volume of water intake. Micturition events were characterized by large urine stain areas on the absorbent pads, often located in a corner of the cage. The average number of daily voiding events at baseline (2.5 ± 0.4) was comparable in both the MM-sham (2.8 ± 0.2) and MM-ES groups (2.6 ± 0.3). Daily water intake for MM-ES group at baseline (160.8 ± 16.3 mL) and during the treatment period (177.4 ± 23.7 mL) were also similar and comparable to those in the MM-sham group (222.9 ± 33.8 mL *vs*. 227.5 ± 18.2 mL; Fig. 3(e)–(h)).

Together, these results indicate that the increase in pad weight observed in the MM-ES group was due to increased voided volume, likely due to the increase in bladder efficiency (i.e., reduced residual volume).

### E. BsN ES Increases Bladder Efficiency and Urethral Closure

**Figure 4.**
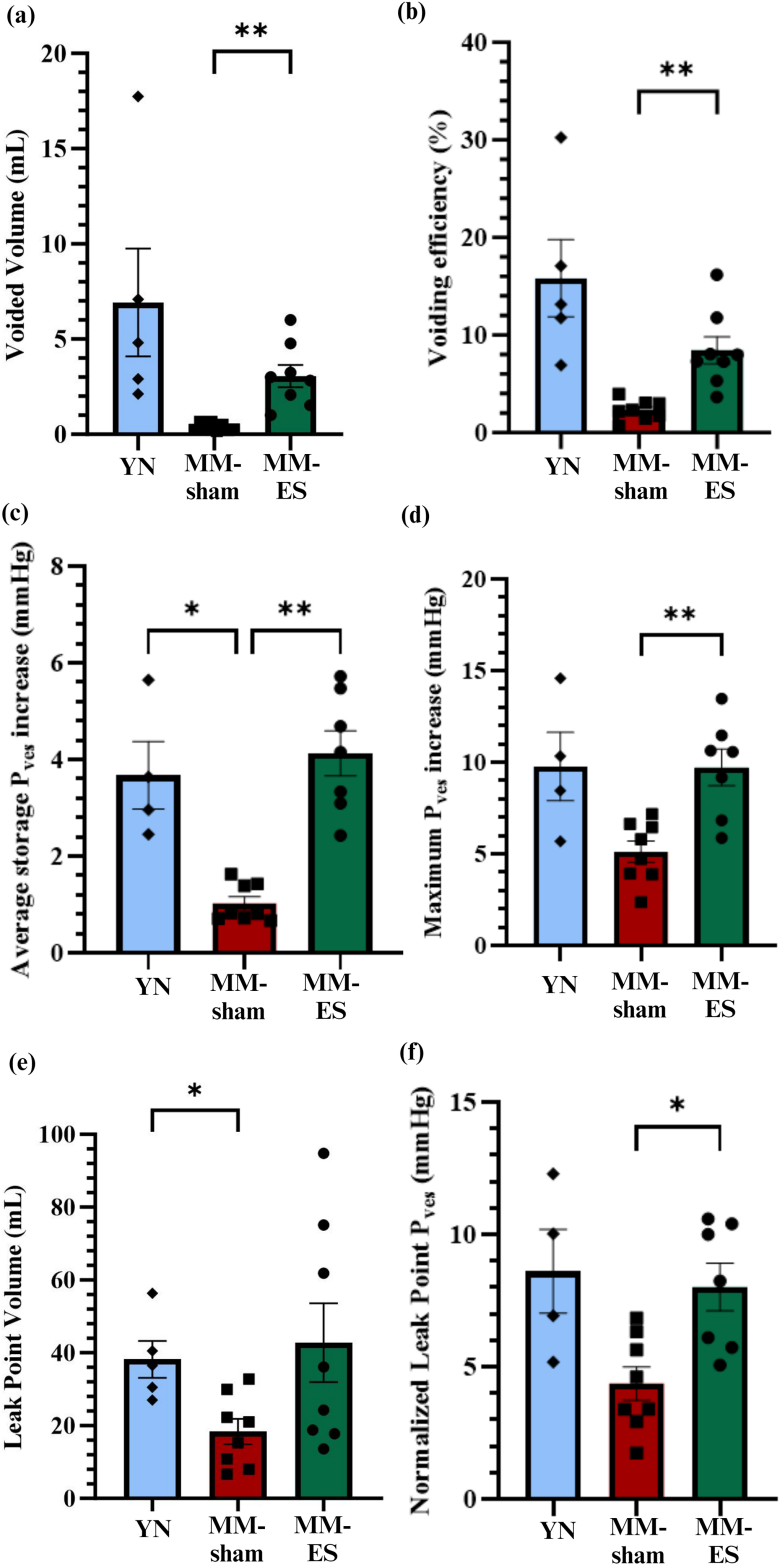
BsN ES increases bladder capacity and urethral closure efficiency. Compared to MM-sham group, MM-ES group has increased (a) voided volume and (b) voiding efficiency, indicating the increased efficiency of BsM; increased (c) average storage P_ves_, (d) maximum P_ves_, and I leak point volume, indicating increased bladder capacity; and (f) increased leak point P_ves_, which indicates increased urethral closure efficiency. All pressure values have been normalized against baseline and averaged for each animal. (YN: n = 5, MM-sham: n = 8, MM-ES: n = 8).

Sham young nulliparous (YN; n=5) not implanted stimulator but exposed to the antenna electrical field, showed an average voided volume of 6.9 ± 2.8 mL, and voiding efficiency of 15.8 ± 4.0%. In contrast, MM-sham rabbits (n=8) showed a drastically reduced voided volume (0.4 ± 0.1 mL) and voiding efficiency (2.3 ± 0.4%; p<0.001), confirming some SUI-related deficits in these animals. Neuromodulation of the BsN in MM-ES treatment group (n=8) resulted in a significant increase in the voided volume compared to the MM-sham group (3.1 ± 0.6 mL, p<0.01), and an increased voiding efficiency of 8.4 ± 1.4%. This suggests improved BsM function in the treated animals, which while significant, only achieved approximately 50% of that in YN controls (Fig. 4(a) and (b)).

We then evaluated the normalized bladder storage pressure (P_ves_) (i.e average P_ves_ during storage – average baseline P_ves_) which in MM-sham group was 1.0 ± 0.1 mmHg, n=8), approximately a third of that in YN group (3.7 ± 0.7 mmHg, n=4, p≤0.03). MM-ES group showed P_ves_ values that were comparable to those of YN group (4.1 ± 0.5 mmHg) and significantly improved compared to MM-sham group (n=7, p≤0.002; Fig. 4(c)). Similarly, the maximum P_ves_ increase during storage (i.e., maximum P_ves_ during storage phase – average baseline P_ves_) was higher for YN group (9.8 ± 1.9 mmHg, n=4), compared to the MM-sham group (5.1 ± 0.6 mmHg, n=8), and improved by sub-chronic BsN ES in MM-ES group (9.7 ± 1.0 mmHg, n=7; p< 0.001) (Fig. 4(d)). The increase in bladder storage P_ves_ is an indication of improved bladder capacity.

Next, we evaluated the maximum bladder volume prior to leaking (leak point volume) to directly test the sphincter function of the BsM. YN group achieved a bladder volume of 38.2 ± 5.1 mL (n=5) prior to leaking, while MM-sham group only achieved less than 50% of the normal capacity (18.4 ± 3.5 ml, n=8). Congruently with the behavioral results, BsN neuromodulation reversed that deficit as MM-ES rabbits showed a leak volume of 42.8 ± 10.8 mL (n=8), comparable to that of YN rabbits and significantly improved over MM-sham rabbits (p= 0.03; Fig. 4(e)).

Measurement of the normalized leak point P_ves_ (i.e., leak point P_ves_ – average baseline P_ves_) confirmed these results as YN group showed sustained 8.6 ± 1.6 mmHg pressure before leaking (n=4), which was comparable to that of MM-ES group (8.02 ± 0.90 mmHg, n=7), and significantly better than MM-sham animals (4.4 ± 0.6 mmHg; p<0.02, n=8; Fig. 4(f)).

### F. BsM and Bladder Morphology

**Figure 5.**
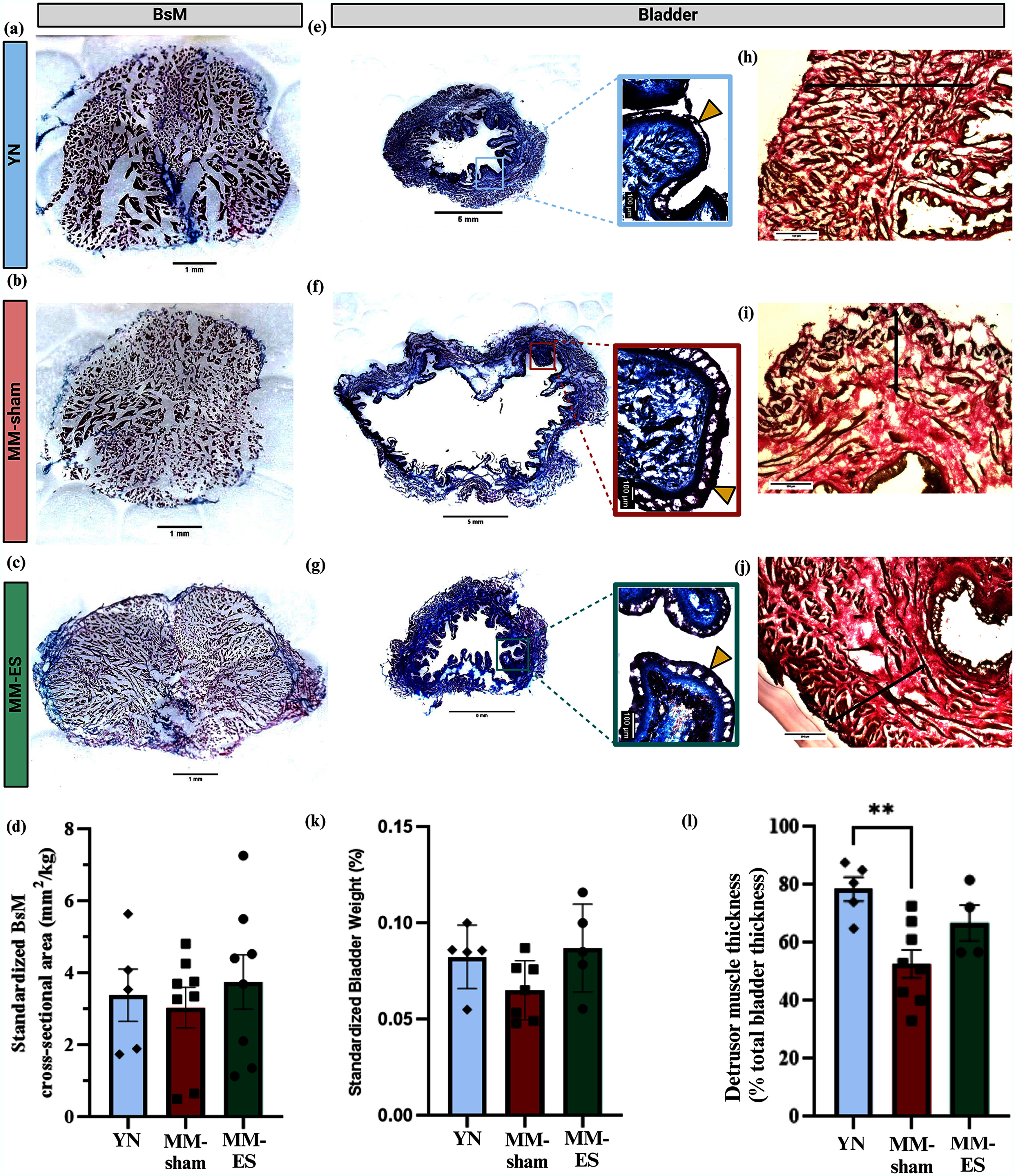
BsM and bladder morphology after BsN ES. Representative images of masson trichrome-stained tissues of medial regions of (a–c) BsM, (e–g) whole bladder, (h–j) modified verhoeff's-stained magnified bladder wall cross-sections for YN, MM-sham, and MM-ES groups (top to bottom). Yellow triangles identify the urothelium layer in the magnified panels and double-ended black arrows demonstrate the detrusor muscle layer thickness in (h–j). Scale bars included. Analyses of (d) standardized medial BsM cross-sectional area, (k) standardized bladder weight, and (l) bladder detrusor muscle layer thickness as percentage of total bladder thickness, comparing YN, MM-ES and MM-sham groups.

The noted improvements in bladder voided volume and bladder efficiency suggested that BsN neuromodulation treatment might have directly strengthened the BsM and indirectly affected the morphology of the bladder. To test this possibilities, we first performed gross morphometry of the medial BsM (Fig. 5(a)–(c)) and found that BsN ES showed an average increase in whole muscle cross-sectional area from 3.0 ± 0.6 mm^2^/kg in MM-sham to 3.7 ± 0.8 mm^2^/kg in MM-ES animals (n=8 each group), which was comparable to that in YN controls (3.4 ± 0.7 mm^2^/kg, n=5), but failed to reach statistical significance (Fig. 5(d)).

Gross morphometry of the bladder was analyzed (Fig. 5(e)–(g)) and standardized bladder weight as a percentage of body weight compared between groups. Bladder weight in the YN control group (0.1 ± 0.01%, n=5) was approximately 34% greater compared to the MM-sham group (0.07 ± 0.01%, n=7), and values in MM-ES animals showed an average increase (0.09 ± 0.01%, n=5) and were comparable to the YN controls (Fig. 5(k)), but not statistically different. Analysis of the bladder detrusor muscle layer (muscularis propria) thickness, quantified as percentage of total bladder thickness (Fig. 5(h)–(j)) showed a significant decrease in the MM-sham group compared to the healthy YN (YN: 78.1 ± 4.1 %, n=5 *vs* MM-sham: 52.7 ± 4.9%, n=8; p= 0.006), which is partially recovered in the MM-ES group, without reaching significance (66.9 ± 6.2, n= 4) (Fig. 5(l)).

## III. Discussion

Acute neuromodulation of the BsN in mature (4-5 years of age) multiparous rabbits has been shown to improve urethral closure and bladder efficiency [18], [19]. This study optimized the use of standard VSA [20] to demonstrate for the first time a behavioral SUI-like deficit of 25–100% leak frequency in retired breeder female rabbits, which were mature (2–3 years of age) and with an average of 11 parities, confirming the use of this animal model for the study of SUI-like function.

We also provided evidence of the efficacy of a 2-minute active BsN ES three times a week, that significantly reduce leak events by 40%. Additionally, in the MM-ES experimental group, BsN-ES resulted in a significantly increased total voided volume, with no significant reduction in the voiding frequency or significant changes in water intake. Given that the water intake and number of voids is similar, the additional voided volume likely comes from an increased voided efficiency (14 g/void, approximately 25% of baseline), which seemed not sufficient to dramatically affect the number of daily voids in the female rabbit model.

Current findings on increased daily voided volume, voiding efficiency, and leak point P_ves_ after the BsN ES treatment indicate an improvement in BsM function, bladder efficiency and urethral closure. It should be noted that the use of propofol as general anesthetic during cystometry has been reported to suppress the micturition reflex, and at high doses (1 mg/kg/min) reduces bladder contraction, voiding efficiency, and EUS EMG activity [21]. However, at lower doses, similar to those in our study, consistent detrusor reflex and maximum urethral closure pressure is preserved [22], [23], [24]. Additionally, as all animals underwent cystometry at standard anesthetic conditions, the results are then comparable across groups.

The role of the perineal and pelvic floor muscles for pelvic organ support and urethral closure, and the deficiencies that contribute to urinary incontinence, are only partially understood [25], [26]. This study shows evidence that sub-chronic stimulation of BsN strengthens the BSM, a perineal muscle reported to be partially damaged by aging and multiparity in rabbits [18]. Neuromodulation of pelvic and perineal muscles can be used to enhance their pelvic organ support function and their secondary sphincter role, thus assisting in urethral closure and partially reversing SUI-like deficits. In-depth understanding of these muscle functions is crucial for designing advanced and targeted treatment options for SUI. In addition to the direct efferent effect on BsM, indirect effect of the BsN stimulation on bladder reflex and CNS plasticity should also be considered as a mechanism of action for the behavioral and functional changes observed [27], [28].

Further histological and molecular analysis will be required to better understand the mechanisms that play a role in the plasticity of the BsM and of the bladder urothelium and detrusor muscles observed. While this study demonstrated the benefit of sub-chronic ES in improving SUI-like deficits in mature multiparous rabbits, chronic studies are necessary to explore whether beneficial treatment effects persist after a short treatment period, and to refine our understanding of the possible long-term mechanisms of action responsible for these changes.

The current wNClip design is sufficient for activation of the BsN through ≤2 cm. However, in humans, the BsM does not seem to play a significant role in urethral closure. Rather, the pelvic floor muscles including the pubococcygeus muscle have been shown to be often damaged and functionally altered by multiparity and aging. Therefore, the nerve target in humans likely include those in the PFM that form the proximal sphincter, and possibly include the pudendal nerve that is known to control the external urethral sphincter [29]. Since these nerves are larger and deeper in humans, the wNClip will need to be appropriately scaled, and the power and control systems enhanced to allowed for the wireless communication, likelyincluding an increase in the size and number of turns of the copper wire in the receiver antenna.

## IV. Conclusion

Overall, our findings demonstrate that unilateral, targeted sub-chronic neuromodulation of BsN can significantly improve SUI-like deficits in MM rabbits, including both behavioral parameters (leak frequency and daily micturition), and functional parameters (voided volume, voiding efficiency, leak point pressure and volume, maximum storage P_ves_, bladder capacity and urethral closure efficiency). Thus, this BsN neuromodulation treatment paradigm has potential use in the treatment of SUI.

## V. Materials and Methods

### A. Neural Electrode Characterization

A miniaturized, fully implantable wireless electrode NeuroClip (wNClip) (RBI Medical) with a slide-and-lock mechanism was used for targeted neuromodulation of the BsN (Fig. 1(a)). The device is powered by electromagnetic induction using an eternal antenna with a 6.78 MHz resonant frequency (Fig. 1(b)). *In vitro* functional testing was conducted in 29 devices to evaluate induction power range and linear and angular current outputs at variable distance and compared against the BsN thresholds determined acutely in MM animals (n=7) (see supplementary materials section 1.1).

### B. Animal Use

All animal experiments were approved by the University of Houston Animal Care Operations (IACUC protocol PROTO202000016) and in accordance with AAALAC and ARRIVE guidelines.

**Figure 6.**
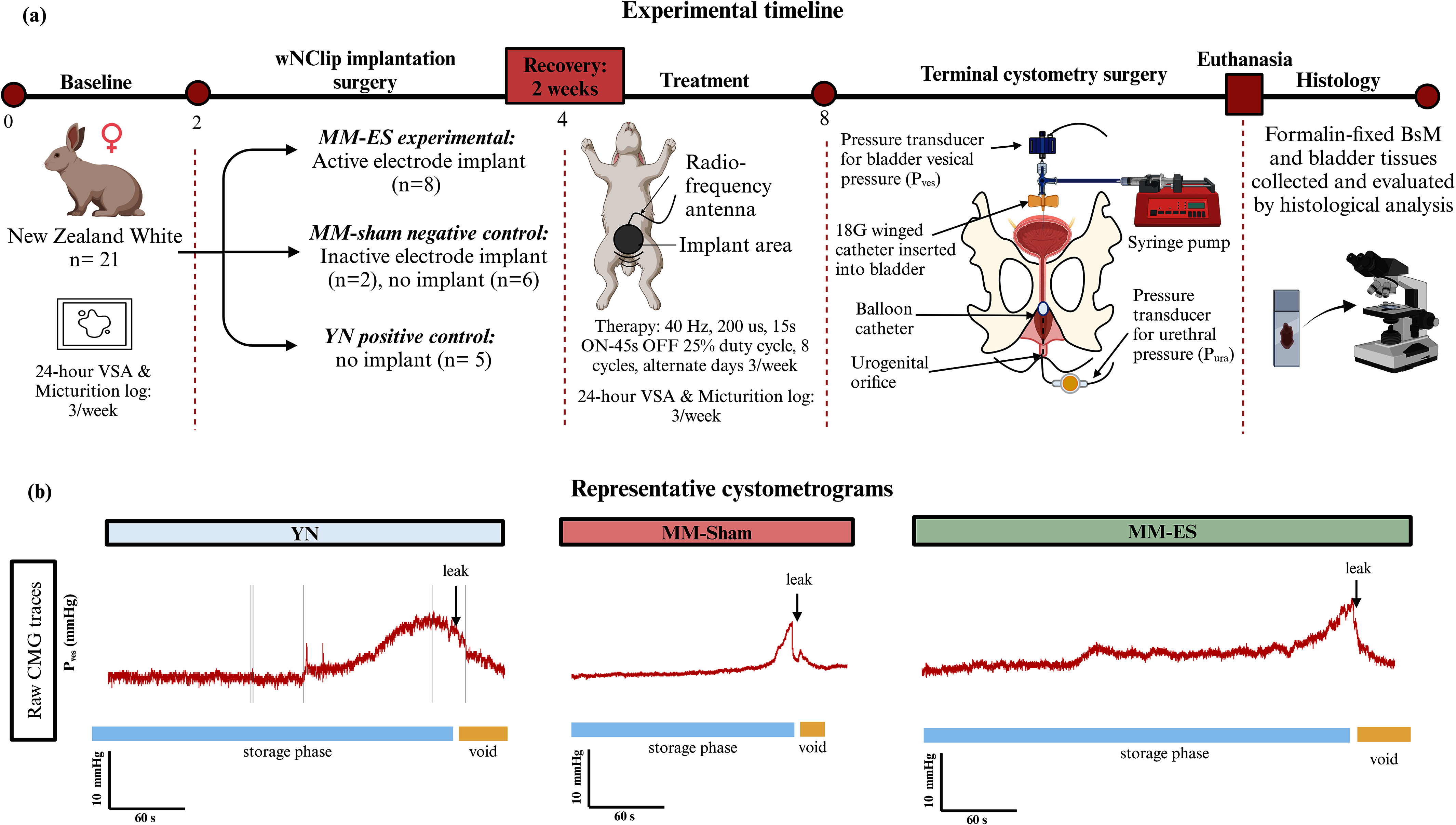
Research methods. (a) Experimental timeline. (b) Representative raw CMG graphs obtained during cytometry.

A total of 21 New Zealand White female rabbits were used in this study, with n=16 being mature multiparous (MM) (27.3 ± 4.59 months, 3.93 ± 0.322 kg, 10.93 ± 3.59 parities) and n=5 being positive control young nulliparous (YN) (5.8 ± 3.83 months, 3.15 ± 0.382 kg, 0 deliveries) (Charles Rivers, Envigo). MM animals were randomly sorted into MM-ES experimental (n=8) and MM-sham negative control (n=8) groups. Researchers were blinded to the animal groups while conducting data collection, treatment, and quantitative analysis. Fig. 6 shows a summary of the study design and timeline. See supplementary materials section 1.3 for details.

### C. VSA and Micturition Analysis

Rabbit micturition data and water intake were recorded during baseline and treatment periods, taken over 24-hour periods, 3 days/week to record leak events, daily micturition mass, daily voiding frequency and daily water intake (Table 1; supplementary materials section 1.4).

**TABLE 1:** Micturition Analysis Parameters and Formulae

Parameter	Description	Formula
Daily micturition (g)	Total mass of micturition collected in a 24-hour window	(wet weight - dry weight)_daytime_ + (wet weight – dry weight)_overnight_
Daily voiding frequency	Total number of voids identified from pad image analysis in a 24-hour window	Void_daytime_ + Void_overnight_
Average micturition per void (g)	Average mass per micturition event	$\frac{{total\ daily\ micturition\ mass}}{{total\ daily\ voids}}$
Daily water intake (mL)	Animal water intake volume in a 24-hour window	Water volume_final_ – Water volume_initial_
Leak event frequency (%)	Animal leak event incidence frequency over time	$\frac{{number\ of\ leak\ events}}{{number\ of\ days\ analyzed}} \times 100\% $
		

For the VSA analysis, wet pad images were analyzed to determine the number of voids in the pad, and leak events were identified using the following criteria: 1) the spot must be less than 3 cm in diameter to differentiate leak spots from small voids; and 2) the spot must be away from an active void to avoid misidentification of leak events from void splatters.

### D. Electrode Implantation

Survival surgery was conducted as described in supplementary materials section 1.4. The BsM, and then BsN, were exposed, validated, and then implanted with wNClip. During implantation, the BsN was first aligned over the wNClip, and then slid through the narrow Z-shaped insertion chamber, undergoing a ≤20% acute elongation, and into the nerve chamber, where it regained its original shape and was ‘locked’ in contact with the electrodes. The implant was validated using a short 15-s electrical stimulation and evaluating associated muscle response, secured in place using Kwik-cast, and the incision was closed.

MM animals were randomly sorted into MM-ES experimental (n=8) and MM-sham negative control (n=8) groups. Active electrodes (validated through bench top testing and *in vitro* during implantation) were implanted in the MM-ES animals (n=8). Sham electrodes (n=2) or no implants (n=6) were placed in the MM-sham group, and no implants were placed in the YN positive control group (n=5).

### E. Neuromodulation Treatment

After a 2-week recovery period following the survival surgery, all animals underwent a 4-week standardized treatment procedure under awake conditions, as described in supplementary materials section 1.6. Treatment was provided on alternate days 3 times per week, with stimulation parameters set at 40-Hz and 200 μs, which had been optimized in a previous study [19] and is within normal safety standards for chronic ES [30], [31], [32]. Each treatment session consisted of 8 cycles of 15-s stimulation train followed by 45-s rest period (25% duty cycle) to minimize fatigue. This provides a 120s effective stimulation of BsM, which mimics the daily BsM contraction achieved during voiding [33]. The alternate day treatment schedule has been shown to improve muscle function without adverse effect from fatigue [34], [35] and therefore was selected for this comparatively high-intensity treatment paradigm. While all animals underwent the treatment protocol, neuromodulation of BsN was achieved only in the MM-ES group which had active implants, while the MM-sham and YN animals did not have implants or had non-functional implants.

### F. Terminal Bladder Catheterization and Urodynamic Set-Up

After the 4-week treatment period was completed, animals underwent non-survival surgery during which functional cystometry was conducted, as described in supplementary materials section 1.7. The bladder was exposed, catheterized, and connected to the urodynamic system.

After cystometry cycles were completed, the animals were administered heparin (I.V., 24 I.U./kg) to prevent blood coagulation and euthanized using pentobarbital (I.V., 120 mg/kg). Cardiovascular perfusion was immediately performed after euthanasia, using 0.9% saline for flushing and 4% paraformaldehyde solution for tissue fixation. BsM and whole bladder were collected, placed in 4% paraformaldehyde solution for 3 days at 4 °C, and then stored in 1X phosphate buffered saline (PBS) at 4 °C.

### G. Functional Cystometry Analysis

Cystometry was conducted by filling the bladder with warm 0.9% saline solution at a constant rate of 2 mL/min until one voiding event was completed. Simultaneous P_ves_ and urethral pressure (P_ura_) data was recorded using the LabChart software (Fig. 6(b); see supplementary materials section 1.8). At least 3 cystometry cycles, when possible, were conducted for each animal, with a 10-minute resting period between each cycle. A 30-s baseline P_ves_ and P_ura_ was also recorded before the filling for each cycle was started.

A custom MATLAB program was used to post-process (data detrending and filtering using a 5-Hz Butterworth filter), and to analyze the data providing the following functional parameters: the average baseline P_ves_, average P_ves_ during the storage phase of the cystometry cycle, the leak point pressure (LPP), and the maximum P_ves_ achieved during storage phase. All P_ves_ values during filling were corrected for the syringe pump pressure. Table 2 summarizes the functional parameters evaluated.

**TABLE 2:** Cystometry Parameters and Formulae

Parameter	Description	Formula
Leak Point Volume (mL)	Volume in the bladder at which leak occurs	$Volume\ infused\ from\ pump\ at\ which\ leak\ is\ observed + volume\ retained\ in\ bladder$ $from\ previous\ cycle$
Voided volume (mL)	Volume expelled during a leak event/void	Directly collected during void event
Voiding efficiency (%)	Percentage of volume voided during leak event/void	$\frac{{voided\ volume}}{{leak\ point\ volume}}\ \times 100\% $
Average storage P_ves_ increase (mmHg)	Average change in P_ves_ during the storage phase compared to baseline	$Corrected\ average\ storage\ {{P}_{ves}}$ $\ - average\ baseline\ {{P}_{ves}}$
Normalized Leak point pressure (mmHg)	Change in P_ves_ at which leak occurs compared to baseline	$Corrected\ leak\ point\ {{P}_{ves}}\ - average\ baseline\ {{P}_{ves}}$
Maximum P_ves_ increase	Maximum change in P_ves_ during storage phase compared to baseline	$Corrected\ maximum\ {{P}_{ves}}\ - average\ baseline\ {{P}_{ves}}$

### H. BsM and Bladder Histology

Collected tissues were dissected to remove surrounding fascia. After dissection, each cleaned bladder was weighed. The BsM and medial bladder tissues were cryoprotected consecutively in 10%, 20% and 30% sucrose solutions, and flash frozen in O.C.T. compound (Tissue-Tek). A cryostat (Epredia CryoStar NX50) was used to obtain 30-µm frozen cross-sections of the medial area of BsM (muscle mid-point), and 20-μm frozen cross-sections of the medial area of the bladder. The frozen sections were stained using Masson Trichrome (Sigma-Aldrich HT15) and modified Verhoeff's elastin stain kit (ScyTek Laboratories ETS1) and imaged under the microscope (Leica S9i, EVOS M5000). ImageJ software was then used to quantify BsM cross-sectional area and bladder muscle layer thickness (see supplementary section 1.7).

### I. Statistical Analysis

Statistical analysis was conducted using the GraphPad Prism software 10.0.1 for Windows (GraphPad Software, San Diego, CA, USA). The ROUT method (Q=1) was used to exclude outliers. Data distribution was tested for normality using Shapiro-wilk test (α= 0.05). Descriptive analysis was conducted to calculate data mean, median, standard deviation (SD) and standard error of mean (SEM). To compare leak events and micturition analysis results between baseline and treatment periods within each group, two-tailed paired t-test was conducted for normally distributed data sets and Wilcoxon test was used for non-normal data sets. To compare micturition data between MM-ES experimental and MM-sham negative control groups, two-tailed unpaired Welch's t-test was conducted for normal data sets and Mann-Whitney test was conducted for non-normal data sets. Functional cystometry data was compared between the experimental, negative control and positive control groups using Brown-Forsythe and Welch one-way ANOVA with multiple comparison for normally distributed data sets, and Kruskal-Wallis test with multiple comparisons for non-normal distribution data. Results are reported as mean ± SEM. Statistical significance: *p < 0.05, **p <0.01, ***p <0.005, ****p < 0.0001.

## Author Contributions

**FSR:** design of experiments, surgeries, animal handling, data acquisition, quantification and analysis, data interpretation, figures preparation, manuscript preparation and revision. **ZY:** animal handling, data acquisition and quantification. **FC** and **MM-G**: conception of research and manuscript review. **PZ:** conception of research, interpretation of data, and manuscript review. **MA** and **YMA:** interpretation of data and manuscript review. **MR-O:** conception of research, design of experiments, surgeries, manuscript preparation and revision. All authors contributed to the article and approved the submitted version.

## Conflict of Interest

MR-O owns shares in Juniper Biomedical, a medical device company. FSR is currently an intern at Juniper Biomedical but was not affiliated with the company during the study and data analysis period. Juniper Biomedical did not have any role in animal data collection, analysis, or in the manuscript.

## Supplementary Materials

Supplementary materials
