# Supplementary material for: Neuromodulation Improves Stress Urinary Incontinence-Like Deficits in Female Rabbits
Source: IEEE Open J Eng Med Biol. 2024 Jun 3;6:10–9. doi: 10.1109/OJEMB.2024.3408454 (PMC11573403; doi:10.1109/OJEMB.2024.3408454)
Supplement: Supplementary materials [file supp1-3408454.docx]

Neuromodulation Improves Stress Urinary Incontinence-like Deficits in Female Rabbits

**F. S. Rahman^1^, Z. Yousuf^2^, F. Castelán ^3,4^, M. Martínez-Gómez^3,4^, Y. M. Akay^1^ (Senior Member, IEEE), P. Zimmern^4^, M. Akay^1^ (Life Fellow, IEEE), M. I. Romero-Ortega^2*^ (Member, IEEE)**

^1^University of Houston, Houston, TX, USA

^2^University of Arizona, Tucson, AZ, USA

^3^Centro Tlaxcala de Biología de la Conducta, Universidad Autónoma de Tlaxcala, Tlaxcala, México

^4^Universidad Nacional Autonoma de México, Instituto de Investigaciones Biomédicas, Unidad Foránea Tlaxcala, Tlaxcala, México

^5^University of Texas Southwestern Medical Center, Dallas, TX, USA

CORRESPONDING AUTHOR: Mario I. Romero-Ortega (e-mail: romeroortega@arizona.edu)

This work was supported in part by the U.S. National Institutes of Health under Grant NIH 1 R01 DK120307-01.

This article has supplementary downloadable material.

**Abstract**  *Objective:* Stress urinary incontinence (SUI) affects a third of the female population and is characterized by involuntary urine leakage during abdominal efforts such as sneezing, laughing, or coughing. Acute neuromodulation of the bulbospongiosus nerve (BsN) was shown to increase bladder efficiency in aged and multiparous rabbits. This study investigates the efficacy of sub-chronic BsN neuromodulation in alleviating SUI-like deficits in mature multiparous rabbits, characterized by increased urine leakage and reduced leak point pressure*. Results:* Using the voiding spot assay, we observed a 40% reduction in urine leakage events after 30 days of BsN stimulation, which correlated with a 60% increase in daily micturition volume, a 10-fold increase in voided volume, and improvements in voiding efficiency and leak point pressure compared to negative control animals. *Conclusion:* In multiparous rabbits, BsN neuromodulation improves important SUI-like metrics including bladder capacity and urethral closure, supporting the use of this bioelectronic modality as treatment for SUI.

**Index Terms** bioelectronics, electrical stimulation, pelvic floor disorders, pelvic innervation, neural interfaces

**Impact Statement** Sub-chronic neuromodulation of the bulbospongiousus nerve results in significant improvement of SUI-like deficits in multiparous rabbits, supporting the notion that chronic stimulation of perineal nerves represents a possible therapeutic option for women suffering from SUI.

INTRODUCTION^^[[1]](#footnote-1)^^

S

tress urinary incontinence (SUI) is a condition characterized by involuntary urine leakage during sudden increases in intra-abdominal pressure, such as during jumping, sneezing, laughing, or coughing [1]. Pelvic floor muscles (PFMs) provide mechanical support to pelvic organs and proximally wrap around the urethra as part of the perineal complex, assisting in urethral closure [2]. These muscles act as a secondary sphincter during sudden increases in abdominal pressure, assisting the external urinary sphincter (EUS) and contributing to the “guardian reflex” to prevent leakage [3]. Weakened PFMs contribute to SUI, which affect approximately 20-35% of adult women, which result from several factors, including pregnancy, parity, and aging [4, 5, 6, 7, 8, 9, 10].

Animals models of SUI include aging and/or multiparous female rabbits, which show anatomical and physiological signs of weakened PFM, deficient urethral closure, and reduced bladder capacity. [11, 12, 13, 14, 15, 16, 17]. This animal model also shows partial nerve damage of the pelvic and perineal nerves including the pubococcygeus nerve (PcN) and bulbospongiosus nerve (BsN) [18, 19]. We previously reported that acute electrical stimulation (ES) of the BsN in multiparous rabbits effectively recruits the bulbospongiosus muscle (BsM), increasing the maximum urethral closure pressure and partially reversing the deficits on bladder efficiency and urethral closure [18, 19]. However, the long-term efficacy of this therapy had not been previously investigated.

In this study, we developed a miniaturized implantable neural stimulator and evaluated the effect of a 30-day wireless neuromodulation of the BsN in mature multiparous (MM) female rabbits. SUI-like deficits such as spontaneous urine leakage, daily voided volume, and leak point pressure were investigated. We observed a decrease in SUI-like deficits, supporting the use of this bioelectronic approach as a potential treatment for SUI.

Results

*Wireless neural stimulator evokes BsM contraction*

Twenty-nine miniature wireless NeuroClip (wNClip) stimulator devices (4.7 x 4.0 x 1.7 mm^3^; Fig. 1a-b) were fabricated by Juniper Biomedical (see supplemental materials 1.1). The device uses a slide-and-lock mechanism to clip onto the BsN. The wNClip is implanted by placing it underneath the target nerve, aligned over the insertion channel, and gently and briefly (i.e. for 15-30 s) lifting the device such that the nerve elongates, reducing its diameter (≤20%), and allowing it to pass through the narrow Z-shaped channel that is designed to prevent easy dislodgment. Once in the electrode chamber, releasing the pull on the nerve allows it to regain its original shape, thereby locking the nerve in place (Fig. 1d).

For stimulation, we use cathodic first bi-phasic electrical pulses powered by electromagnetic induction with an external antenna. A threshold current of 0.1 mA efficiently evoked the contraction of the BsM (see supplementary materials 1.2). The angular distance from the antenna sufficient to power the device and depolarize the BsN was evaluated. Current output was measured at 1 to 5 cm and angles of 0º, 30º and 60º (Fig. 1e). A maximum of 0.23 mA was measured at 1 cm from the antenna. Effective BsM contraction was obtained at up to 2 cm and 30º angle form the antenna (Fig. 1f).


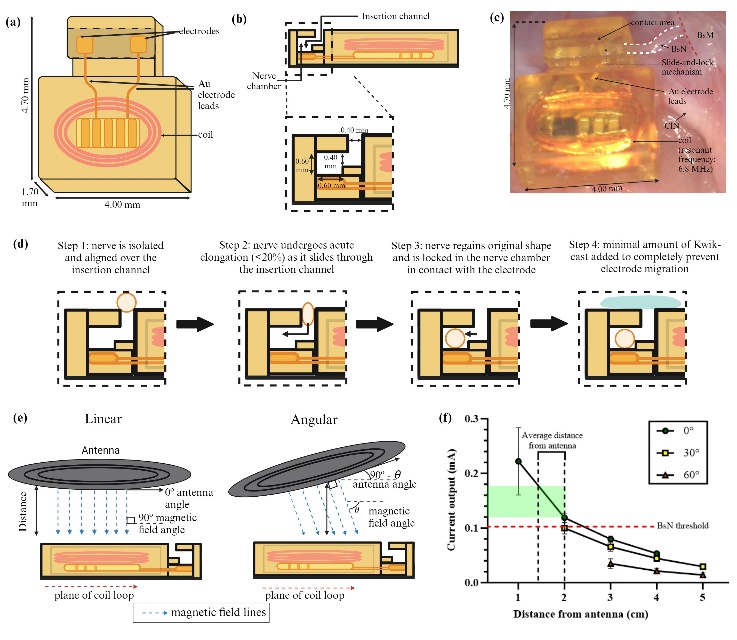
**Fig. 1. wNClip implantation and function.** (a-b) Device schematic (c) Picture of device on the BsN. (d) Schematic of the ‘slide-and-lock’ mechanism of wNClip implantation. (e) Angular test for powering the wNClip using the external antenna. (f) Current output as a function of antenna distance and angle of 29 wNClip devices. Shaded green area shows targeted current output.

*BsN stimulation reduces leak events*

Leak events were determined as urine spots smaller than 3 mm on absorbent pads placed under the animal cage floor, monitored for 24-hours, three times per week. A 2-week baseline period was established, followed by a 4-week BsN ES treatment period. This voiding spot assay (VSA) showed that young nulliparous (YN) rabbits (n=5) did not leak during the study, whereas mature multiparous sham rabbits (MM-sham, n=8) leaked 25-75% of the time. Sham ES (i.e., inactive electrodes or animals with no implant but exposed to the antenna pulsed electrical field) showed no change in the percentage of daily leak events. In contrast, mature multiparous rabbits that received neuromodulation treatment (MM-ES, n=8) showed a significant (p<0.0001) decrease in leak frequency, from 69.4 ± 8.2% at baseline, to 29.4 ± 5.2% during the treatment. This represented a 40.0 ± 4.5% reduction in leak frequency (Fig. 2b-c). The baseline values of both MM-ES and MM-sham group were comparable (p=0.09). These results demonstrated that BsM neuromodulation significantly reduced leak events, a metric associated with SUI.


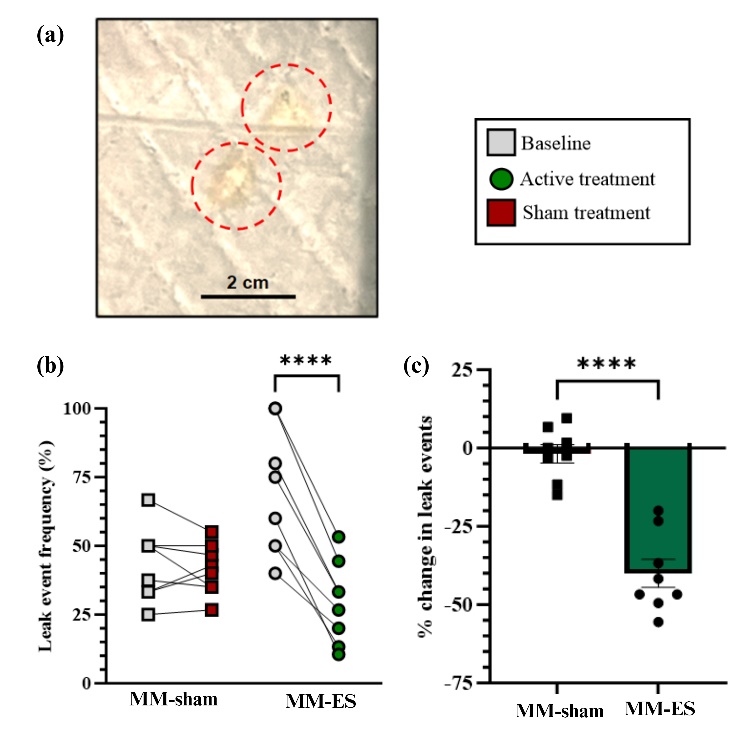
**Fig. 2. BsN ES reduces leak frequency**. (a) Representative image of leak event identified on the absorbent pad. MM-ES group (n=8) showed a significant decrease in leak event frequency (b) between baseline and treatment periods and, (c) from the negative control MM-sham group (n=8).

*BsN ES increases micturition volume*

To evaluate if voided volume changed during micturition over time, we measured the pad weight 3 times a week and compared the values at baseline and during ES treatment. MM-ES animals showed a 33.1 ± 8.0 g (60.7%) increase in total daily voided volume compared to pre-treatment levels (54.5 ± 7.5g at baseline *vs.* 87.6 ± 8.3 g during treatment; p=0.01; n=8). Pad weight values in MM-sham animals did not change significantly (93.8 ± 17.7 g at baseline *vs.* 75.6 ± 13.9 g during treatment, p=0.2; Fig. 3a-b). The baseline values for the MM-ES and MM-sham groups were comparable (p= 0.2).

A similar trend was observed for the average micturition per void in MM-ES group, which showed a significant increase of 14.3 ± 2.4 g (p= 0.003) between baseline and stimulation periods (24.8 ± 4.3 g *vs*. 39.1 ± 3.5 g, p≤ 0.0005). Conversely, MM-sham rabbits showed similar values at baseline (36.8 ± 6.7 g) and treatment period (30.7 ± 6.4 g; p≤0.2) (Fig. 3c-d).

*BsN ES does not affect voiding frequency or daily water intake*

We then evaluated if the increase in pad weight was due to an increase in the number of micturition events or an increased volume of water intake. Micturition events were characterized by large urine stain areas on the absorbent pads, often located in a corner of the cage. The average number of daily voiding events at baseline (2.5 ± 0.4) was comparable in both the MM-sham (2.8 ± 0.2) and MM-ES groups (2.6 ± 0.3). Daily water intake for MM-ES group at baseline (160.8 ± 16.3 mL) and during the treatment period (177.4 ± 23.7 mL) were also similar and comparable to those in the MM-sham group (222.9 ± 33.8 mL *vs*. 227.5 ± 18.2 mL; Fig. 3e-h).

Together, these results indicate that the increase in pad weight observed in the MM-ES group was due to increased voided volume, likely due to the increase in bladder efficiency (i.e., reduced residual volume).


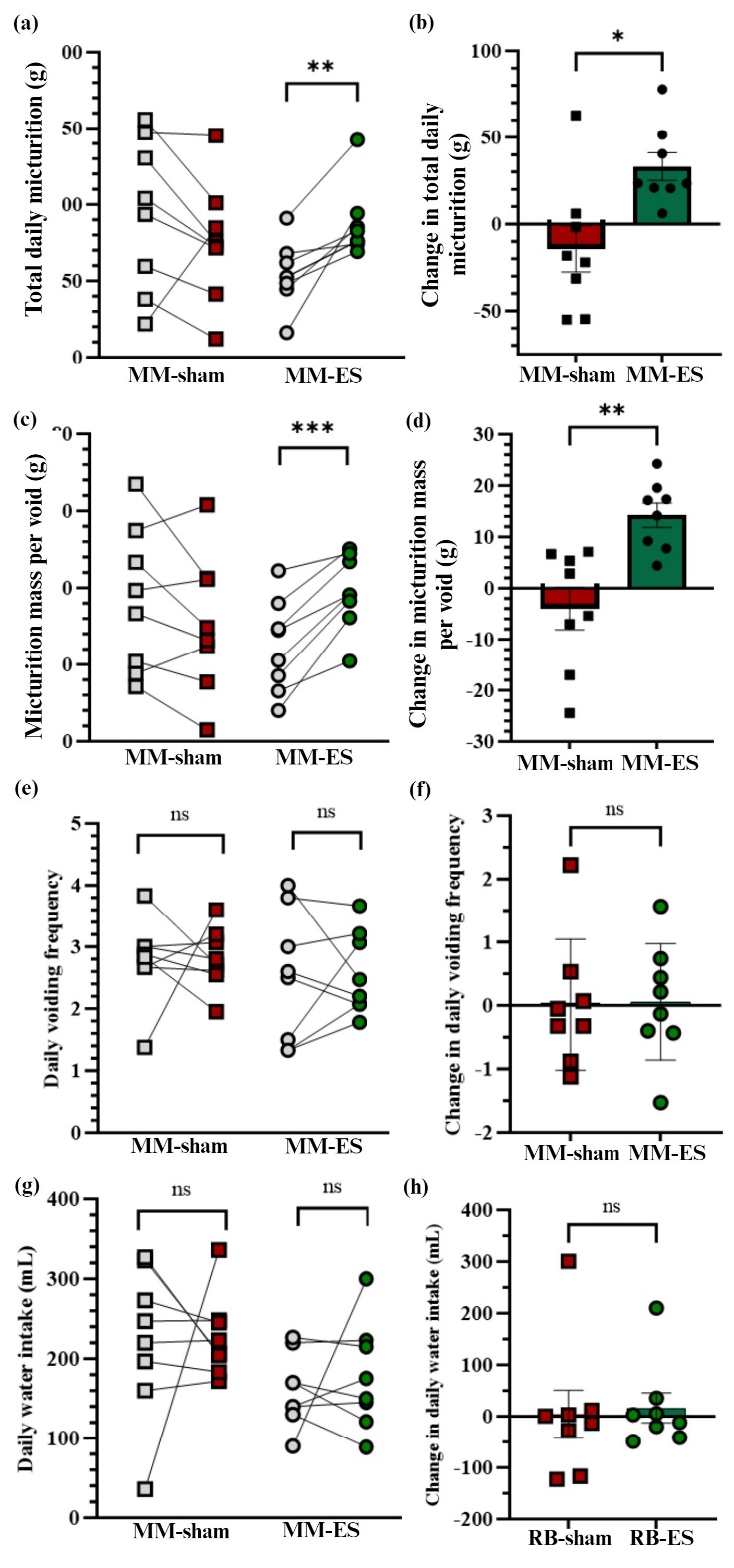


**Fig. 3.** **BsN ES achieved increased daily voided volume.** MM-ES group showed increased (a-b) daily micturition and (c-d) increased average micturition per void. No significant differences were found between MM-ES and MM-sham groups for (e-f) voiding frequency and (g-h) daily water intake.

*BsN ES increases bladder efficiency and urethral closure*

Sham young nulliparous (YN; n=5) not implanted stimulator but exposed to the antenna electrical field, showed an average voided volume of 6.9 ± 2.8 mL, and voiding efficiency of 15.8 ± 4.0%. In contrast, MM-sham rabbits (n=8) showed a drastically reduced voided volume (0.4 ± 0.1 mL) and voiding efficiency (2.3 ± 0.4%; p<0.001), confirming some SUI-related deficits in these animals. Neuromodulation of the BsN in MM-ES treatment group (n=8) resulted in a significant increase in the voided volume compared to the MM-sham group (3.1 ± 0.6 mL, p<0.01), and an increased voiding efficiency of 8.4 ± 1.4%. Suggesting improved BsM function in the treated animals, which while significant, only achieved approximately 50% of that in YN controls (Fig. 4a-b).

We then evaluated the normalized bladder storage pressure (P_ves_) (i.e average P_ves_ during storage – average baseline P_ves_) which in MM-sham group was 1.0 ± 0.1 mmHg, n=8), approximately a third of that in YN group (3.7 ± 0.7 mmHg, n=4, p≤0.03). MM-ES group showed P_ves_ values that were comparable to those of YN group (4.1 ± 0.5 mmHg) and significantly improved compared to MM-sham group (n=7, p≤0.002; Fig 4c). Similarly, the maximum P_ves_ increase during storage (i.e. maximum P_ves_ during storage phase – average baseline P_ves_) was higher for YN group (9.8 ± 1.9 mmHg, n=4), compared to the MM-sham group (5.1 ± 0.6 mmHg, n=8), and improved by sub-chronic BsN ES in MM-ES group (9.7 ± 1.0 mmHg, n=7; p< 0.001) (Fig. 4d). The increase in bladder storage P_ves_ is an indication of improved bladder capacity.

Next, we evaluated the maximum bladder volume prior to leaking (leak point volume) to directly test the sphincter function of the BsM. YN group achieved a bladder volume of 38.2 ± 5.1 mL (n=5) prior to leaking, while MM-sham group only achieved less than 50% of the normal capacity (18.4 ± 3.5 ml, n=8). Congruently with the behavioral results, BsN neuromodulation reversed that deficit as MM-ES rabbits showed a leak volume of 42.8 ± 10.8 mL (n=8), comparable to that of YN rabbits and significantly improved over MM-sham rabbits (p= 0.03; Fig 4e).

Measurement of the normalized leak point P_ves_ (i.e., leak point P_ves_ – average baseline P_ves_) confirmed these results as YN group showed sustained 8.6 ± 1.6 mmHg pressure before leaking (n=4), which was comparable to that of MM-ES group (8.02 ± 0.90 mmHg, n=7), and significantly better than MM-sham animals (4.4 ± 0.6 mmHg; p<0.02, n=8; Fig. 4f).


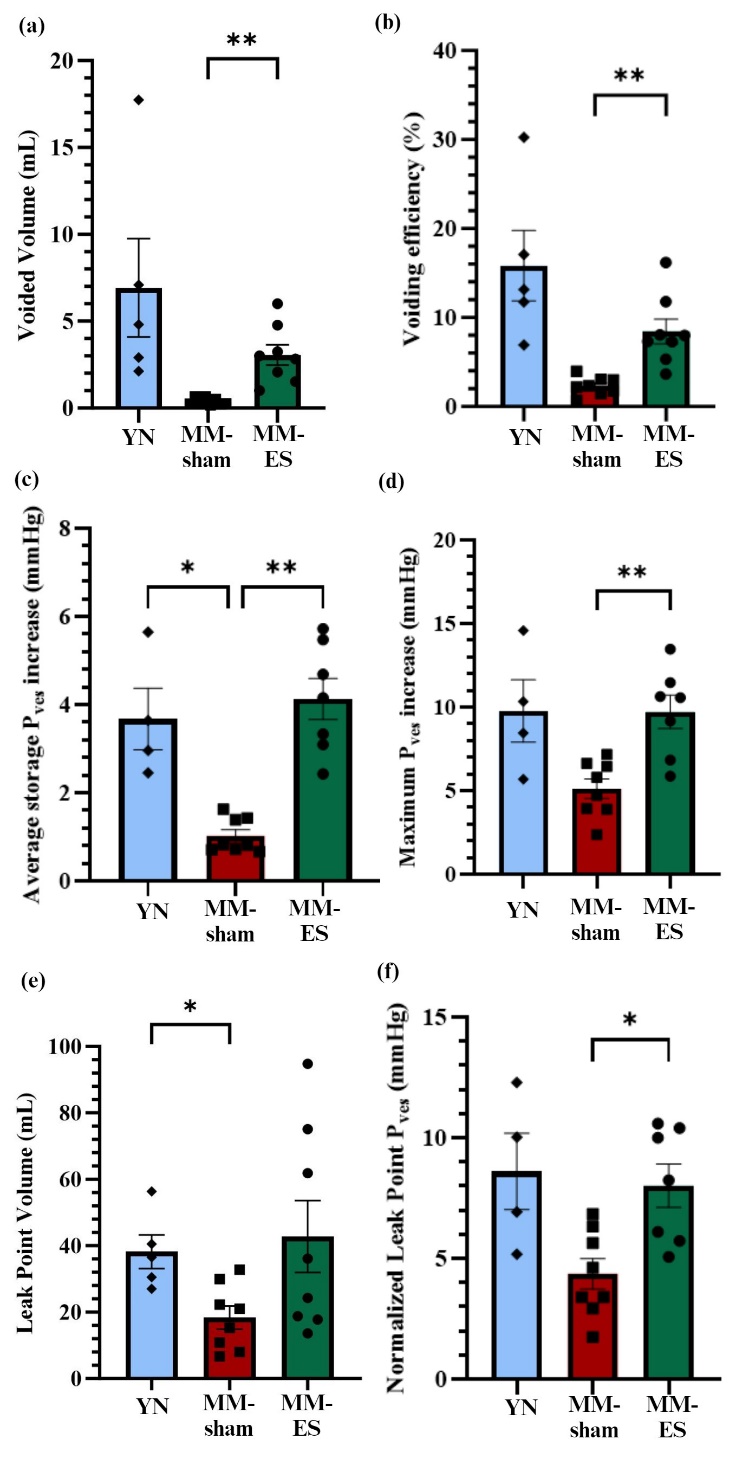
**Fig. 4. BsN ES increases bladder capacity and urethral closure efficiency.** Compared to MM-sham group, MM-ES group has increased (a) voided volume and (b) voiding efficiency, indicating the increased efficiency of BsM; increased (c) average storage P_ves_, (d) maximum P_ves_, and I leak point volume, indicating increased bladder capacity; and (f) increased leak point P_ves_, which indicates increased urethral closure efficiency. All pressure values have been normalized against baseline and averaged for each animal. (YN: n=5, MM-sham: n=8, MM-ES: n=8)

*BsM and bladder morphology*

The noted improvements in bladder voided volume and bladder efficiency suggested that BsN neuromodulation treatment might have directly strengthened the BsM and indirectly affected the morphology of the bladder. To test this possibilities, we first performed gross morphometry of the medial BsM (Fig. 5a-c) and found that BsN ES showed an average increase in whole muscle cross-sectional area from 3.0 ± 0.6 mm^2^/kg in MM-sham to 3.7 ± 0.8 mm^2^/kg in MM-ES animals (n=8 each group), which was comparable to that in YN controls (3.4 ± 0.7 mm^2^/kg, n=5), but failed to reach statistical significance (Fig. 5d).

Gross morphometry of the bladder was analyzed (Fig. 5e-g) and standardized bladder weight as a percentage of body weight compared between groups. Standardized bladder weight in the YN control group (0.1 ± 0.01%, n=5) was approximately 34% greater compared to the MM-sham group (0.07 ± 0.01%, n=7), and values in MM-ES animals showed an average increase (0.09 ± 0.01%, n=5) and were comparable to the YN controls (Fig. 5k), but not statistically different. Analysis of the bladder detrusor muscle layer (muscularis propria) thickness, quantified as percentage of total bladder thickness (Fig 5h-j) showed a significant decrease in the MM-sham group compared to the healthy YN (YN: 78.1 ± 4.1 %, n=5 *vs* MM-sham: 52.7 ± 4.9%, n=8; p= 0.006), which is partially recovered in the MM-ES group, without reaching significance (66.9 ± 6.2, n= 4) (Fig 5l).


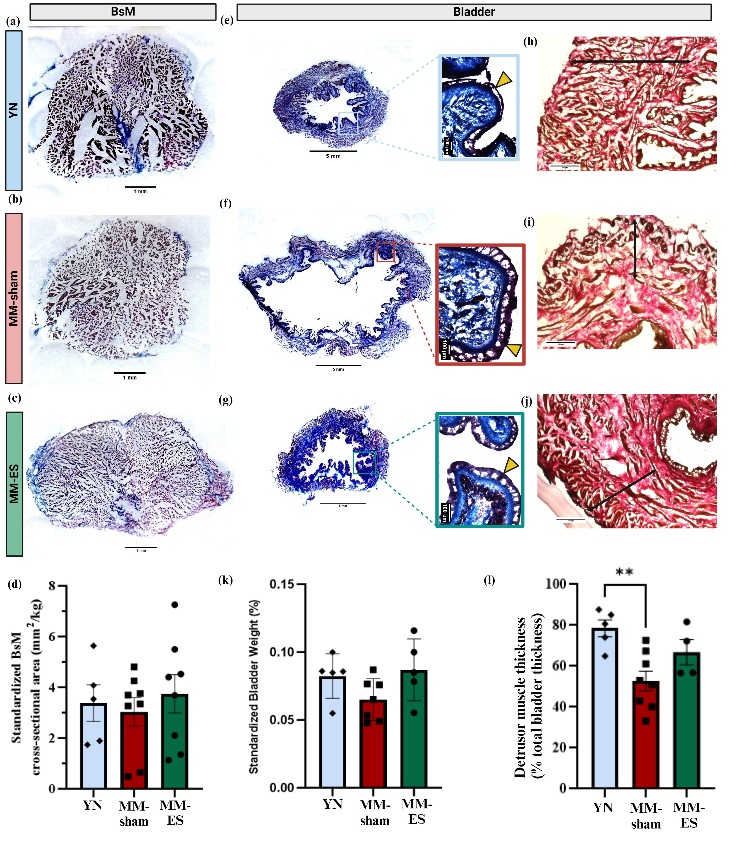


**Fig. 5.** **BsM and bladder morphology after BsN ES**. Representative images of Masson trichrome-stained tissues of medial regions of (a-c) BsM, and (e-g) whole bladder, and (h-j) modified Verhoeff’s-stained magnified bladder wall cross-sections for YN, MM-sham, and MM-ES groups (top to bottom). Yellow triangles identify the urothelium layer in the magnified panels and double-ended black arrows demonstrate the detrusor muscle layer thickness in (h-j). Scale bars included. Analyses of (d) standardized medial BsM cross-sectional area, (k) standardized bladder weight, and (l) bladder detrusor muscle layer thickness as percentage of total bladder thickness, comparing YN, MM-ES and MM-sham groups.

Discussion

Acute neuromodulation of the BsN in mature (4-5 years of age) multiparous rabbits has been shown to improve urethral closure and bladder efficiency [18, 19]. This study optimized the use of standard VSA [20] to demonstrate for the first time a behavioral SUI-like deficit of 25-100% leak frequency in retired breeder female rabbits, which were mature (2-3 years of age) and with an average of 11 parities, confirming the use of this animal model for the study of SUI-like function.

We also provided evidence of the efficacy of a 2-minute active BsN ES three times a week, to significantly reduce leak events by 40%. Additionally, in the MM-ES experimental group, BsN-ES resulted in a significantly increased total voided volume, with no significant reduction in the voiding frequency or significant changes in water intake. Given that the water intake and number of voids is similar, the additional voided volume likely comes from an increased voided efficiency (14 g/void, approximately 25% of baseline), which seemed not sufficient to dramatically affect the number of daily voids in the female rabbit model.

Current findings on increased daily voided volume, voiding efficiency, and leak point P_ves_ after the BsN ES treatment indicate an improvement in BsM function, bladder efficiency and urethral closure. It should be noted that the use of propofol as general anesthetic during cystometry has been reported to suppress the micturition reflex, and at high doses (1 mg/kg/min) reduces bladder contraction, voiding efficiency, and EUS EMG activity [21]. However, at lower doses, similar to those in our study, consistent detrusor reflex and maximum urethral closure pressure is preserved [22] [23] [24]. Additionally, as all animals underwent cystometry at standard anesthetic conditions, the results are comparable across groups.

The role of the perineal and pelvic floor muscles for pelvic organ support and urethral closure, and the deficiencies that contribute to urinary incontinence, are only partially understood [25, 26]. This study shows evidence that sub-chronic stimulation of BsN strengthens the BSM, a perineal muscle reported to be partially damaged by aging and multiparity in rabbits [18]. Neuromodulation of pelvic and perineal muscles can be used to enhance their pelvic organ support function and their secondary sphincter role, thus assisting in urethral closure and partially reversing SUI-like deficits. In-depth understanding of these muscle functions is crucial for designing advanced and targeted treatment options for SUI. In addition to the direct efferent effect on BsM, indirect effect of the BsN stimulation on bladder reflex and CNS plasticity should also be considered as a mechanism of action for the behavioral and functional changes observed [27] [28].

Further histological and molecular analysis will be required to better understand the mechanisms that play a role in the plasticity of the BsM and of the bladder urothelium and detrusor muscles observed. While this study demonstrated the benefit of sub-chronic ES in improving SUI-like deficits in mature multiparous rabbits, chronic studies are necessary to explore whether beneficial treatment effects persist after a short treatment period, and to refine our understanding of the possible long-term morphological mechanisms of action responsible for these changes.

The current wNClip design is sufficient for activation of the BsN through ≤2 cm. However, in humans, the BsM does not seem to play a significant role in urethral closure. Rather, the pelvic floor muscles including the pubococcygeus muscle have been shown to be often damaged and functionally altered by multiparity and aging. Therefore, the nerve target in humans is likely to be those in the PFM that form the proximal sphincter, and the pudendal nerve that is known to control the external urethral sphincter [29]. Since these nerves are larger and deeper in humans, the wNClip will need to be appropriately adapted in size, and the power and control systems enhanced to allowed for the wireless communication, this will include an increase in the size and number of turns of the copper wire in the receiver antenna.

Conclusions

Overall, our findings demonstrate that unilateral, targeted sub-chronic neuromodulation of BsN can significantly improve SUI-like deficits in MM rabbits, including both behavioral parameters (leak frequency and daily micturition), and functional parameters (voided volume, voiding efficiency, leak point pressure and volume, maximum storage P_ves_, bladder capacity and urethral closure efficiency). Thus, this BsN neuromodulation treatment paradigm has potential use in the treatment of SUI.

Materials and Methods

*Neural electrode characterization*

A miniaturized, fully implantable wireless electrode NeuroClip (wNClip) (RBI Medical) with a slide-and-lock mechanism was used for targeted neuromodulation of the BsN (Fig. 1a). The device is powered by electromagnetic induction using an eternal antenna with a 6.78 MHz resonant frequency (Fig.1b). *In vitro* functional testing was conducted in 29 devices to evaluate induction power range and linear and angular current outputs at variable distance and compared against the BsN thresholds determined acutely in MM animals (n=7) (see supplementary materials section 1.1).

*Animal Use*

All animal experiments were approved by the University of Houston Animal Care Operations (IACUC protocol PROTO202000016) and in accordance with AAALAC and ARRIVE guidelines.

A total of 21 New Zealand White female rabbits were used in this study, with n=16 being mature multiparous (MM) (27.3 ± 4.59 months, 3.93 ± 0.322 kg, 10.93 ± 3.59 parities) and n=5 being positive control young nulliparous (YN) (5.8 ± 3.83 months, 3.15 ± 0.382 kg, 0 deliveries) (Charles Rivers, Envigo). MM animals were randomly sorted into MM-ES experimental (n=8) and MM-sham negative control (n=8) groups. Researchers were blinded to the animal groups while conducting data collection, treatment, and quantitative analysis. Fig. 6 shows a summary of the study design and timeline. See supplementary materials section 1.3 for details.


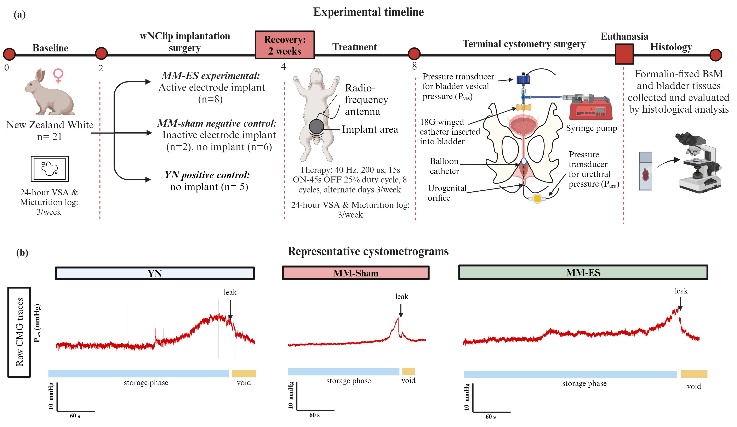


**Fig. 6. Research Methods.** (a) Experimental timeline. (b) Representative raw CMG graphs obtained during cytometry.

*VSA and Micturition analysis*

Rabbit micturition data and water intake were recorded during baseline and treatment periods, taken over 24-hour periods, 3 days/week to record leak events, daily micturition mass, daily voiding frequency and daily water intake (Table I; supplementary materials section 1.4).

For the VSA analysis, wet pad images were analyzed to determine the number of voids in the pad, and leak events were identified using the following criteria: (1) the spot must be less than 3 cm in diameter to differentiate leak spots from small voids; and (2) the spot must be away from an active void to avoid misidentification of leak events from void splatters.

TABLE I

Micturition Analysis Parameters and Formulae

| **Parameter** | **Description** | **Formula** |
| --- | --- | --- |
| Daily micturition (g) | Total mass of micturition collected in a 24-hour window | (wet weight - dry weight)_daytime_ + (wet weight – dry weight)_overnight_ |
| Daily voiding frequency | Total number of voids identified from pad image analysis in a 24-hour window | Void_daytime_ + Void_overnight_ |
| Average micturition per void (g) | Average mass per micturition event | $\frac{total daily micturition mass}{total daily voids}$ |
| Daily water intake (mL) | Animal water intake volume in a 24-hour window | Water volume_final_ – Water volume_initial_ |
| Leak event frequency (%) | Animal leak event incidence frequency over time | $\frac{number of leak events}{number of days analyzed}\times100\%$ |
|  |  |  |

*Electrode implantation*

Survival surgery was conducted as described in supplementary materials section 1.4. The BsM, and then BsN, were exposed, validated, and then implanted with wNClip. During implantation, the BsN was first aligned over the wNClip, and then slid through the narrow Z-shaped insertion chamber, undergoing a ≤20% acute elongation, and into the nerve chamber, where it regained its original shape and was ‘locked’ in contact with the electrodes. The implant was validated using a short 15-s electrical stimulation and evaluating associated muscle response, secured in place using Kwik-cast, and the incision was closed.

MM animals were randomly sorted into MM-ES experimental (n=8) and MM-sham negative control (n=8) groups. Active electrodes (validated through bench top testing and *in vitro* during implantation) were implanted in the MM-ES animals (n=8). Sham electrodes (n=2) or no implants (n=6) were placed in the MM-sham group, and no implants were placed in the YN positive control group (n=5).

*Neuromodulation treatment*

After a 2-week recovery period following the survival surgery, all animals underwent a 4-week standardized treatment procedure under awake conditions, as described in supplementary materials section 1.6. Treatment was provided on alternate days 3 times per week, with stimulation parameters set at 40-Hz and 200 μs, which had been optimized in a previous study [19] and is within normal safety standards for chronic ES [30, 31, 32]. Each treatment session consisted of 8 cycles of 15-s stimulation train followed by 45-s rest period (25% duty cycle) to minimize fatigue. This provides a 120s effective stimulation of BsM, which mimics the daily BsM contraction achieved during voiding [33]. The alternate day treatment schedule has been shown to improve muscle function without adverse effect from fatigue [34, 35] and therefore was selected for this comparatively high-intensity treatment paradigm. While all animals underwent the treatment protocol, neuromodulation of BsN was achieved only in the MM-ES group which had active implants, while the MM-sham and YN animals did not have implants or had non-functional implants.

*Terminal bladder catheterization and urodynamic set-up*

After the 4-week treatment period was completed, animals underwent non-survival surgery during which functional cystometry was conducted, as described in supplementary materials section 1.7. The bladder was exposed, catheterized, and connected to the urodynamic system.

After cystometry cycles were completed, the animals were administered heparin (I.V., 24 I.U./kg) to prevent blood coagulation and euthanized using pentobarbital (I.V., 120 mg/kg). Cardiovascular perfusion was immediately performed after euthanasia, using 0.9% saline for flushing and 4% paraformaldehyde solution for tissue fixation. BsM and whole bladder were collected, placed in 4% paraformaldehyde solution for 3 days at 4ºC, and then stored in 1X phosphate buffered saline (PBS) at 4ºC.

*Functional cystometry analysis*

Cystometry was conducted by filling the bladder with warm 0.9% saline solution at a constant rate of 2 mL/min until one voiding event was completed. Simultaneous P_ves_ and urethral pressure (P_ura_) data was recorded using the LabChart software (Fig 6b; see supplementary materials section 1.8). At least 3 cystometry cycles, when possible, were conducted for each animal, with a 10-minute resting period between each cycle. A 30-s baseline P_ves_ and P_ura_ was also recorded before the filling for each cycle was started.

A custom MATLAB program was used to post-process (data detrending and filtering using a 5-Hz Butterworth filter), and to analyze the data providing the following functional parameters: the average baseline P_ves_, average P_ves_ during the storage phase of the cystometry cycle, the leak point pressure (LPP), and the maximum P_ves_ achieved during storage phase. All P_ves_ values during filling were corrected for the syringe pump pressure. Table II summarizes the functional parameters evaluated.

TABLE II

Cystometry Parameters and Formulae

| **Parameter** | **Description** | **Formula** |
| --- | --- | --- |
| Leak Point Volume (mL) | Volume in the bladder at which leak occurs | $Volume infused from pump at which leak is observed+volume retained in bladder$  $from previous cycle$ |
| Voided volume (mL) | Volume expelled during a leak event/void | Directly collected during void event |
| Voiding efficiency (%) | Percentage of volume voided during leak event/void | $\frac{voided volume}{leak point volume} \times100\%$ |
| Average storage P_ves_ increase (mmHg) | Average change in P_ves_ during the storage phase compared to baseline | $Corrected average storage P_{ves}$  $-average baseline P_{ves}$ |
| Normalized Leak point pressure (mmHg) | Change in P_ves_ at which leak occurs compared to baseline | $Corrected leak point P_{ves} -average baseline P_{ves}$ |
| Maximum P_ves_ increase | Maximum change in P_ves_ during storage phase compared to baseline | $Corrected maximum P_{ves} -average baseline P_{ves}$ |

*BsM and Bladder Histology*

Collected tissues were dissected to remove surrounding fascia. After dissection, each cleaned bladder was weighed. The BsM and medial bladder tissues were cryoprotected consecutively in 10%, 20% and 30% sucrose solutions, and flash frozen in O.C.T. compound (Tissue-Tek). A cryostat (Epredia CryoStar NX50) was used to obtain 30-µm frozen cross-sections of the medial area of BsM (muscle mid-point), and 20-μm frozen cross-sections of the medial area of the bladder. The frozen sections were stained using Masson Trichrome (Sigma-Aldrich HT15) and modified Verhoeff’s elastin stain kit (ScyTek Laboratories ETS1) and imaged under the microscope (Leica S9i, EVOS M5000). ImageJ software was then used to quantify BsM cross-sectional area and bladder muscle layer thickness (see supplementary section 1.7).

*Statistical analysis*

Statistical analysis was conducted using the GraphPad Prism software 10.0.1 for Windows (GraphPad Software, San Diego, CA, USA). The ROUT method (Q=1) was used to exclude outliers. Data distribution was tested for normality using Shapiro-wilk test (α= 0.05). Descriptive analysis was conducted to calculate data mean, median, standard deviation (SD) and standard error of mean (SEM). To compare leak events and micturition analysis results between baseline and treatment periods within each group, two-tailed paired t-test was conducted for normally distributed data sets and Wilcoxon test was used for non-normal data sets. To compare micturition data between MM-ES experimental and MM-sham negative control groups, two-tailed unpaired Welch’s t-test was conducted for normal data sets and Mann-Whitney test was conducted for non-normal data sets. Functional cystometry data was compared between the experimental, negative control and positive control groups using Brown-Forsythe and Welch one-way ANOVA with multiple comparison for normally distributed data sets, and Kruskal-Wallis test with multiple comparisons for non-normal distribution data. Results are reported as mean ± SEM. Statistical significance: **p <* 0.05*, **p* <0.01, ****p* <0.005, *****p* < 0.0001.

Supplementary Materials

Please see the attached supplementary materials for further details of materials and methods.

Author Contributions

**FSR:** design of experiments, surgeries, animal handling, data acquisition, quantification and analysis, data interpretation, figures preparation, manuscript preparation and revision. **ZY:** animal handling, data acquisition and quantification. **FC** and **MM-G**: conception of research and manuscript review. **PZ:** conception of research, interpretation of data, and manuscript review. **MA** and **YMA:** interpretation of data and manuscript review. **MR-O:** conception of research, design of experiments, surgeries, manuscript preparation and revision. All authors contributed to the article and approved the submitted version.

Acknowledgment

Authors thank Bret See, Yifan Wang, Hoang Tran and Laura Rubio for their technical assistance with the experiments conducted in this study.

All graphical illustrations and figures were created using GraphPad Prism 10.1.01 and Biorender.com respectively.

Conflict of interest

MR-O owns shares in Juniper Biomedical, a medical device company. FSR is currently an intern at Juniper Biomedical but was not affiliated with the company during the study and data analysis period. Juniper Biomedical did not have any role in animal data collection, analysis, or in the manuscript.

References

| [1] | H. T. Bernard, D. de Ridder, F. M. Robert, S. E. Swift, B. Berghmans, J. Lee, A. Monga, E. Petri, D. E. Rizk, P. K. Sand and G. N. Schaer, "An International Urogynecological Association(IUGA)/International Continence Society (ICS) Joint Reporton the Terminology for Female Pelvic Floor Dysfunction," *Neurourology and Urodynamics,* pp. 4-20, 2010. |
| --- | --- |
| [2] | J. DeLancey, F. Pipitone, M. Masteling, B. Xie, J. Ashton-Miller and L. Chen, "Functional Anatomy of Urogenital Hiatus Closure: the Perineal Complex Triad Hypothesis," *Int Urogynecol J.,* vol. 35, no. 2, pp. 441-449, 2024. |
| [3] | J. Park, D. Bloom and E. McGuire, "The Guarding Reflex Revisited," *British Journal of Urology,* vol. 80, no. 6, pp. 940-945, 1997. |
| [4] | U. J. Lee, L. Feinstein, J. B. Ward, Z. Kirkali, E. E. Martinez-Miller, B. R. Matlaga and K. C. Kobashi, "Prevalence of Urinary Incontinence among a Nationally Representative Sample of Women, 2005-2016: Findings from the Urologic Diseases in America Project.," *The Journal of urology,* vol. 205, no. 6, pp. 1718-1724, 2021. |
| [5] | E. Rubilotta, M. Balzarro, A. D'Amico, M. Cerruto, S. Bassi, C. Bovo, V. Iacovelli, D. Bianchi, W. Artibani and E. Finazzi Agrò, "Pure stress urinary incontinence: analysis of prevalence, estimation of costs, and financial impact," *BMC Urol,* vol. 19, no. 1, p. 44, 4 Jun 2019. |
| [6] | K. Falah-Hassani, J. Reeves, R. Shiri, D. Hickling and L. McLean, "The pathophysiology of stress urinary incontinence: a systematic review and meta-analysis," *Int Urogynecol J,* vol. 32, no. 3, pp. 501-552, 2021. |
| [7] | A. R. Smith, G. L. Hosker and D. W. Warrell, "The role of pudendal nerve damage in the aetiology of genuine stress incontinence in women," *British journal of obstetrics and gynaecology,* vol. 96, no. 1, p. 29–32, 1989. |
| [8] | G. Giraudet, L. Patrouix, C. Fontaine, X. Demondion, M. Cosson and C. Rubod, "Three dimensional model of the female perineum and pelvic floor muscles," *European Journal of Obstetrics &amp; Gynecology and Reproductive Biology,* vol. 226, pp. 1-6, 2018. |
| [9] | A. Devreese, F. Staes, W. De Weerdt, H. Feys, A. Van Assche, F. Penninckx and R. Vereecken, "Clinical evaluation of pelvic floor muscle function in continent and incontinent women," *Neurourology and urodynamics,* vol. 23, no. 3, p. 190–197, 2004. |
| [10] | I. C. Lakovschek, G. Trutnovsky, B. Obermayer-Pietsch and D. Gold, "Longitudinal Study of Pelvic Floor Characteristics Before, During, and After Pregnancy in Nulliparous Women," *Journal of ultrasound in medicine : official journal of the American Institute of Ultrasound in Medicine,* vol. 41, no. 1, p. 147–155, 2022. |
| [11] | F. Castelán, K. López-García, S. Moreno-Pérez, R. Zempoalteca, D. Corona-Quintanilla, M. Romero-Ortega, I. Jiménez-Estrada and M. Martínez-Gómez, "Multiparity affects conduction properties of pelvic floor nerves in rabbits.," *Brain Behav,* vol. 8, no. 10, p. e01105, Oct 2018 . |
| [12] | M. Martínez-Gómez, G. Mendoza-Martínez, D. L. Corona-Quintanilla, V. Fajardo, J. Rodríguez-Antolín and F. Castelán, "Multiparity causes uncoordinated activity of pelvic- and perineal-striated muscles and urodynamic changes in rabbits," *Reproductive sciences,* vol. 18, no. 12, p. 1246–1252, 2011. |
| [13] | D. L. Corona-Quintanilla, F. Castelán, V. Fajardo, J. Manzo and M. Martínez-Gómez, "Temporal coordination of pelvic and perineal striated muscle activity during micturition in female rabbits," *J Urol,* vol. 181, no. 3, pp. 1452-8, 2009. |
| [14] | K. López-García, E. Cuevas, O. Sánchez-García, P. Pacheco, M. Martínez-Gómez and F. Castelán, "Differential damage and repair responses of pubococcygeus and bulbospongiosus muscles in multiparous rabbits," *Neurourol. Urodyn.,* vol. 35, no. 2, pp. 180-5, 2016. |
| [15] | D. L. Corona-Quintanilla, R. Zempoalteca, L. Arteaga, F. Castelán and M. Marti­nez-Gomez, "The role of pelvic and perineal striated muscles in urethral function during micturition in female rabbits," *Neurourol Urodyn,* vol. 33, no. 4, pp. 455-60, 2014. |
| [16] | R. Lopez-Juarez, R. Zempoalteca, D. Corona-Quintanilla, I. Jimenez-Estrada, F. Castelan and M. Martonez-Gomez, "Multiparity modifies contractile properties of pelvic muscles affecting the genesis of vaginal pressure in rabbits," *Neurourology and Urodynamics,* vol. 37, pp. 106-114, 2018. |
| [17] | D. Corona-Quintanilla, R. López-Juárez, P. Pacheco, M. Romero-Ortega, F. Castelán and M. Martínez-Gómez, "Bladder and urethral dysfunction in multiparous and mature rabbits correlates with abnormal activity of pubococcygeus and bulbospongiosus muscles," *Neurourol Urodyn,* vol. 39, no. 1, pp. 116-124, 2020. |
| [18] | A. Hernandez-Reynoso, D. Corona-Quintanilla, K. López-García, A. Horbovetz, F. Castelan, P. Zimmern, M. Martinez-Gomez and M. Romero-Ortega, "Targeted neuromodulation of pelvic floor nerves in aging and multiparous rabbits improves continence," *Scientific Reports,* vol. 11, p. 10615, 2021. |
| [19] | A. G. Hernandez-Reynoso, F. S. Rahman, B. Hedden, F. Castelán, M. Martínez-Gómez, P. Zimmern and M. I. Romero-Ortega, "Secondary urethral sphincter function of the rabbit pelvic and perineal muscles," *Frontiers in neuroscience,* vol. 17, p. 1111884, 2023. |
| [20] | W. Hill, M. Zeidel, D. Bjorling and C. Vezina, "Void spot assay: recommendations on the use of a simple micturition assay for mice," *Am J Physiol Renal Physiol,* vol. 315, no. 5, pp. F1422-F1429, 1 November 2018. |
| [21] | A. Moheban, H. Chang and L. Havton, "The Suitability of Propofol Compared with Urethane for Anesthesia during Urodynamic Studies in Rats," *J Am Assoc Lab Anim Sci.,* vol. 55, no. 1, pp. 89-94, 2016. |
| [22] | J. Xu, Z. Yousuf, Z. Ouyang, E. Kennedy, P. Lester, T. Martin and T. Bruns, "Anesthetic agents affect urodynamic parameters and anesthetic depth at doses necessary to facilitate preclinical testing in felines," *Sci Rep,* vol. 10, no. 1, p. 11401, 2020. |
| [23] | T. Cohen, J. Westropp, P. Kass and B. Pypendop, "Evaluation of urodynamic procedures in female cats anesthetized with low and high doses of isoflurane and propofol," *Am J Vet Res,* vol. 70, no. 2, pp. 290-6, 2009. |
| [24] | J. Byron, P. March, S. DiBartola, D. Chew and W. 3. Muir, "Comparison of the effect of propofol and sevoflurane on the urethral pressure profile in healthy female dogs," *Am J Vet Res,* vol. 64, no. 10, pp. 1288-92, 2003. |
| [25] | P. Petros, "THE CASE AGAINST urethral failure is not a critical factor in female urinary incontinence. Now what? The integral theory system," *Neurology and Urodynamics,* vol. 41, no. 6, pp. 1270-1280, 2022. |
| [26] | F. Pipitone, Z. Sadeghi and J. DeLancey, "Urethral function and failure: A review of current knowledge of urethral closure mechanisms, how they vary, and how they are affected by life events," *Neurology and Urodynamics,* vol. 40, no. 8, pp. 1869-1879, 2021. |
| [27] | K. Shapiro, N. Pace, T. Morgan, H. Cai, B. Shen, J. Wang, J. Roppolo, W. de Groat and C. Tai, "Additive Inhibition of Reflex Bladder Activity Induced by Bilateral Pudendal Neuromodulation in Cats," *Front Neurosci,* vol. 14, p. 80, 2020. |
| [28] | S. Brose, D. Bourbeau and K. Gustafson, "Genital nerve stimulation is tolerable and effective for bladder inhibition in sensate individuals with incomplete SCI," *J Spinal Cord Med,* vol. 41, no. 2, pp. 174-181, 2018. |
| [29] | d. G. W. Thor KB, "Neural control of the female urethral and anal rhabdosphincters and pelvic floor muscles," *Am J Physiol Regul Integr Comp Physiol.,* vol. 299, no. 2, pp. R416-38, 2010. |
| [30] | C. Günter, J. Delbeke and M. Ortiz-Catalan, "Safety of long-term electrical peripheral nerve stimulation: review of the state of the art," *J Neuroeng Rehabil.,* vol. 16, no. 1, 2019. |
| [31] | E. Nussbaum, P. Houghton, J. Anthony, S. Rennie, B. Shay and A. Hoens, "Neuromuscular Electrical Stimulation for Treatment of Muscle Impairment: Critical Review and Recommendations for Clinical Practice," *Physiother Can,* vol. 69, no. 5, pp. 1-76, 2017. |
| [32] | D. Merrill, M. Bikson and J. Jefferys, "Electrical stimulation of excitable tissue: design of efficacious and safe protocols," *Journal of Neuroscience Methods,* vol. 141, p. 171–198, 2005. |
| [33] | R. Stein, J. C. Hutcheson, L. Krasnopolsky, D. A. Canning, M. C. Carr and S. A. Zderic, "The decompensated detrusor V," *The Journal of Urology,* vol. 166, no. 2, p. 651–657, August 2001. |
| [34] | J. Agre, A. Rodriquez, T. Franke, E. Swiggum, R. Harmon and J. Curt, "Low-intensity, alternate-day exercise improves muscle performance without apparent adverse affect in post-polio patients," *American Journal of Physical Medicine & Rehabilitation,* vol. 75, no. 1, pp. 50-58, 1996. |
| [35] | B. Doucet, A. Lam and L. Griffin, "Neuromuscular electrical stimulation for skeletal muscle function," *Yale Journal of Biology and Medicine,* vol. 85, no. 2, pp. 201-215, 2012. |
| [36] | J. O. L. DeLancey, "Pelvic Floor Anatomy and Pathology," in *Biomechanics of the Female Pelvic Floor*, Academic Press, 2016, pp. 13-51. |
| [37] | S. Madill and L. McLean, "A Contextual Model of Pelvic Floor Muscle Defects in Female Stress Urinary Incontinence: A Rationale for Physiotherapy Treatment," *Annals of the New York Academy of Sciences,* vol. 1101, pp. 335-360, 2007. |

**Supplementary Materials**

Neuromodulation Improves Stress Urinary Incontinence-like Deficits in Female Rabbits

F.S. Rahman, Z. Yousuf, F. Castelán, M. Martínez-Gómez, Y. M. Akay, P. Zimmern, M. Akay, M. I. Romero-Ortega^*^

1. MATERIALS AND METHODS
   1. *Neural electrode characterization*

wNClip are fabricated using UV-sensitive, medical grade, Epo-TEK Epoxy 301, which is non-toxic and ISO 10993 biocompatible, and encapsulated in biocompatible BIO SLA material (USP Class VI). The electrode circuit consists of a copper wire coil for inductive power, connected to ceramic capacitors, microelectronics, and diodes for rectification (Supplementary fig. 1a). The circuit is bonded to gold electrodes which carry the current to the neural interface contact area. Instead of a magnetic core, the wNClip relies on the miniature circuit that yields a 6-fold increase in energy harvesting and current output. Additionally, this electrode design eliminates the need for a cuff implantation method, which can be cumbersome and not practical for smaller nerves. Instead, the wNClip uses a patent pending “slide-and-lock” nerve attachment mechanism which allows the safe implantation onto small peripheral nerves with relative ease. The device is powered by an external stimulation set-up, consisting of the stimulation antenna, RF amplifier, signal generator and controlled via a customized GUI in a computer (supplementary figure 1b). The GUI is used to set the parameters for stimulation and the external antenna can power the electrode at a resonant frequency of 6.78 MHz.

Accelerated aging tests were conducted for the wNClip using a 0.9% saline bath at 55ºC (Q= 18, ambient 37ºC) for 32 days, which is equivalent to 1 year in the body.

Functional testing for current output was conducted in 29 devices *in vitro* by placing a conductor in the electrode channel and measuring the current across the conductor using a multimeter, at antenna distances 1-5 cm and antenna angles 0-60º.

- 1. *Determination of BsN threshold activation current*

Under anesthetic conditions, the BsM and BsN of the MM rabbits (n=7) were exposed. A bi-polar hook electrode (FHC 30211), connected to a pulse generator (A-M Systems Isolated Pulse Stimulator) was used to directly stimulate the BsN, using 40 Hz, 200 µs pulse width. Stimulation was started at 100 µA, and increased by 50 µA intervals, until any BsM response (muscle ‘twitch’) was observed. The stimulation current was then titrated to the nearest 10 µA to the lowest current at which a muscle response was clearly seen (threshold current).


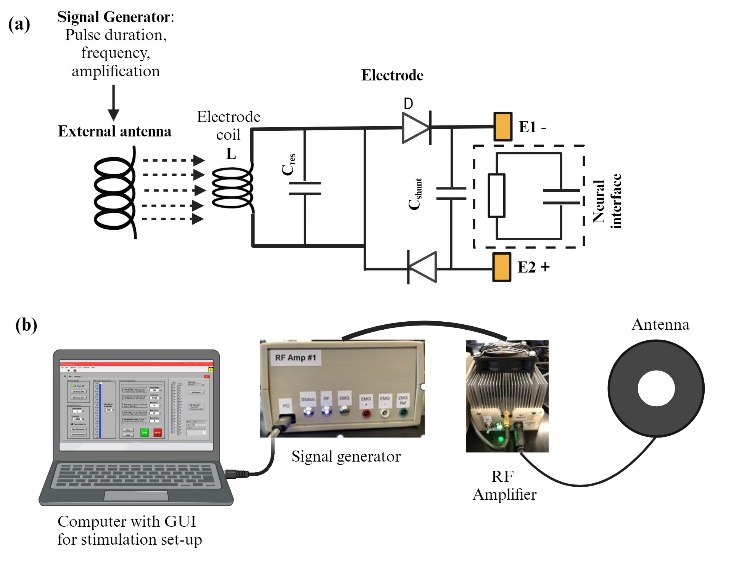


**Supplementary Figure 1.** (a) simplified wNClip circuit. (b) Stimulation setup system for wNClip.

*1.3 Animal use*

All animals (n=21) were habituated for a period of 2 weeks after transportation. The animals were singly housed in Multifloor Pen System Rabbit cage rack (Techniplast) with 12/12-hour light/dark cycle and given Lab Rabbit Diet pellets 5321 with ad libitum access to water via water bottles. After habituation was completed, the animals underwent a 2-week baseline period during which baseline behavioral data was collected, followed by an electrode implantation surgery. Animals were given a 2-week recovery period following the surgery, and a 4-week treatment was conducted afterwards. At the end of the treatment period, animals underwent terminal surgery during which cystometry cycles were conducted to collect urodynamic data. After euthanasia and perfusion, the BsM, BsN and the bladder were collected for histological evaluation.

*1.4 VSA and micturition analysis*

To record the micturition mass, absorbent pads (23x30 inches) were weighed (dry weight) and placed on the waste tray underneath the animal cages. When replacing the pads, the pads were first imaged, the number of voiding spots and any leak events were noted, excess debris and fecal matter were removed, and the used pads were weighed (wet weight). Pads were replaced twice within each 24-hour collection period, with the first pad being placed between 10 AM- 6 PM (daytime collection) and the second pad placed between 6 PM to 10 AM of the next day (overnight collection). Water intake was also analyzed during the 24-hour window by recording the initial and final water volumes in the water bottles. Small aluminum containers were placed under the water bottle spouts to prevent water dripping to the absorbent pad.

*1.5 Electrode implantation*

All survival surgeries were conducted in aseptic conditions using sterilized tools and supplies. Anesthesia was induced using intravenous ketamine (10 mg/kg) + diazepam (0.5 mg/kg) and maintained using isoflurane (0.5-3%). Sustained release buprenorphine (0.01-0.05 mg/kg) was administered intraperitoneally for analgesic effects during the surgical procedure. The surgery area was shaved under anesthesia, and then sanitized using povidone-iodine three times followed by 70% ethanol. The animal was placed in a supine position and a 1-2 cm midline incision on the skin is made between the inferior part of the pubic arch and the rabbit genitals. The fascia beneath the skin was carefully dissected to expose the BsM, which originates from the pubic arch and inserts in the anterior wall of the vagina. The BsM was moved medially and held in place using surgical retractors, exposing the BsN that branches away from the larger clitoral nerve (ClN) and runs perpendicular to the muscle and innervates the muscle at its medial region. Glass rods and micro-scissors were used to dissect the connective tissue underneath, isolating the nerve. Hook electrodes connected to pulse generator (A-M Systems Isolated Pulse Stimulator) were used to stimulate the isolated nerve to validate it and to determine threshold current needed to activate the muscle under 40-Hz and 200 μs stimulation parameters. Once the nerve was validated by visually observing associated muscle response in the BsM, the wNeuroClip was implanted on to the nerve using its slide-and-lock mechanism. The implant was tested using a short 15-s electrical stimulation using a radio-frequency pulse generator (RBI Medical) to ensure proper stimulation by evaluating the associated muscle response. Once validated, the implant was secured in place using a minimal amount of low-toxicity Kwik-cast silicone elastomer. Finally, the skin was sutured close using 4-0 absorbable suture.

The animal was observed continuously for 3 hours after anesthesia was stopped and daily for the following 3 days to ensure proper recovery.

*1.6 Neuromodulation treatment*

During treatment, animals were held loosely by their scruffs in an upright position, with the other hand supporting their back while the handler remained in a seated position. The antenna was placed on the skin above the implant area at a 0º expected angle from the known implant position.

*1.7 Terminal bladder catheterization and urodynamic set-up*

In the terminal surgery, After the 4-week treatment period was completed, animals underwent non-survival surgery during which functional cystometry was conducted. Animal anesthesia was induced and maintained using propofol I.V. (1.5-2.0 mg/kg to induce, 0.2-0.6 mg/kg/min to maintain). Sustained release buprenorphine (0.01-0.05 mg/kg) was administered intraperitoneally for analgesic effects during the surgical procedure. The surgery area was shaved under anesthesia, and then sanitized using povidone-iodine three times followed by 70% ethanol. The animal was placed in a supine position and a 3-4 cm midline incision on the skin was made on the lower abdomen, starting from the superior part of the pubic symphysis upwards. The fascia beneath the skin was dissected to expose the abdominal linea alba, and a 3-4 cm incision was made, separating the muscles. The abdominal fat was carefully dissected and shifted to the sides until the urinary bladder was observed. The bladder was held at the apex using tissue forceps and gently pulled out of the abdominal cavity. 6-0 silk suture was used to make four continuous stitches along the bladder dome (purse-string suture). An 18-G Excel safelet catheter with custom wings placed at the end was inserted into the bladder apex at the center and anchored in place by tightening the purse string suture and suturing through the custom wings. The bladder was replaced into the peritoneal cavity, and the abdominal muscle layer and skin layer were separately sutured back using 4-0 silk sutures.

Urine in the bladder was first extracted using a disposable 10-mL syringe connected to the bladder catheter and then expelled by gently pressing down on the abdominal region. A Foley balloon catheter was advanced 4-cm through the urogenital orifice and under the pelvic bone and inflated using 0.5 mL saline. The disposable syringe was removed from the bladder catheter and both the bladder and urethral catheters were connected to a pre-flushed and pre-calibrated urodynamic system. The urodynamic system consisted of the bladder catheter line connected with both a syringe pump and pressure transducer (MLT0699 Disposable BP Transducer) for simultaneous bladder filling and bladder vesicle pressure (P_ves_) recording, and the urethral catheter line connected to a second pressure transducer (MLT844 Physiological Pressure Transducer) to record urethral pressure (P_ura_).

*1.8 Functional cystometry analysis*

During cystometry, data was collected using LabChart software. Event markers were placed in the software for the start of bladder filling, observation of leak/void from the urogenital orifice (leak point) and end of void. The volume at which leak occurred (leak point volume) and total volume infused into the bladder until end of run were recorded from the syringe pump, and the voided volume was collected using a syringe and recorded. The remaining volume in the bladder was partially expelled manually at the end of each run, and the expelled fluids were collected using a syringe and the extracted volume recorded.

The raw cystometry data was processed using a custom MATLAB code to detrend the data and filter out high frequency noise using a 5-Hz Butterworth filter, and to quantify the following: the average baseline P_ves_, average P_ves_ during the storage phase of the cystometry cycle, the leak point pressure (LPP), and the maximum P_ves_ achieved during storage phase. After the MATLAB processing, all P_ves_ values during filling were corrected for the syringe pump pressure and normalized against baseline to correct for calibration variations.

*1.9 BsM and Bladder Histology*

BsM cross-sectional area was quantified using ImageJ, with each animals having 3 repeated measurements and average calculated, and then standardized BsM cross-sectional area was calculated as follows:

Standardized BsM cross-sectional area (mm^2^/kg) =

$$\frac{average cross-sectional area ({mm}^{2})}{animal weight (kg)}$$

Bladder weight and animal weight was recorded and standardized bladder weight ratio was calculated as follows:

Standardized bladder weight=

$$\frac{bladder weight (kg)}{animal weight \left( kg \right)} \times100\%$$

Using ImageJ, total bladder wall thickness and the thickness of the detrusor muscle layer were measured in triplicates, and the detrusor layer thickness as percentage of total bladder thickness was calculated as follows:

Detrusor layer thickness (% of total bladder wall)

$$\frac{average detrusor thickness}{average total bladder thickness} \times100\%$$

1. [↑](#footnote-ref-1)
